# Parameter estimation in fluorescence recovery after photobleaching: quantitative analysis of protein binding reactions and diffusion

**DOI:** 10.1007/s00285-021-01616-z

**Published:** 2021-06-15

**Authors:** Daniel E. Williamson, Erik Sahai, Robert P. Jenkins, Reuben D. O’Dea, John R. King

**Affiliations:** 1 University of Nottingham, Nottingham, NG7 2RD UK; 2 Tumour Cell Biology Laboratory, The Francis Crick Institute, London, NW1 1AT UK

**Keywords:** Fluorescence recovery after photobleaching, FRAP, Recovery curve, Confocal, Fluorescence microscopy, Reaction–diffusion, 92B05, 92C45, 92E20, 37N25, 35Q92

## Abstract

Fluorescence recovery after photobleaching (FRAP) is a common experimental method for investigating rates of molecular redistribution in biological systems. Many mathematical models of FRAP have been developed, the purpose of which is usually the estimation of certain biological parameters such as the diffusivity and chemical reaction rates of a protein, this being accomplished by fitting the model to experimental data. In this article, we consider a two species reaction–diffusion FRAP model. Using asymptotic analysis, we derive new FRAP recovery curve approximation formulae, and formally re-derive existing ones. On the basis of these formulae, invoking the concept of Fisher information, we predict, in terms of biological and experimental parameters, sufficient conditions to ensure that the values all model parameters can be estimated from data. We verify our predictions with extensive computational simulations. We also use computational methods to investigate cases in which some or all biological parameters are theoretically inestimable. In these cases, we propose methods which can be used to extract the maximum possible amount of information from the FRAP data.

## Introduction

### Fluorescence recovery after photobleaching

The development of live cell fluorescence microscopy has revolutionised molecular cell research. Much modern fluorescence microscopy depends upon the use of the green fluorescence protein (GFP) and its variants. GFP, first isolated from the jellyfish *Aequorea Victoria*, has the ability to absorb energy from light in the ultra violet blue to wavelength range, which is then released by radiating green light (Tsien 1998). By modifying cells to express a fusion of GFP with a particular target protein (tagging or labelling), researchers are able to study gene expression and protein localisation within the living cell (Giepmans et al. 2006). This is done by illuminating the target cell with light of an appropriate wavelength and detecting the green fluorescent emission. However, observation of a cell at steady state reveals little, if anything, about protein mobility. The small size of proteins (way below the resolution limit of light microscopy) and the typically large number of labelled proteins ($10^4{-}10^6$) means that in most experiments it is not possible to follow the movement of individual proteins. Reducing the number of labelled proteins can help to address this problem, but then detecting the fluorescent signal becomes increasingly difficult.

In the 1970s, researchers, mainly Axelrod et al. (2018), began to develop experimental methods to study protein mobility by perturbing the cell under observation. The technique they devised, known as fluorescence recovery after photobleaching (FRAP) (Cone 1972; Poo and Cone 1973; Liebman and Entine 1974; Koppel et al. 1976; Axelrod et al. 1976; Wu et al. 1977) is widely used to this day. Although numerous improvements have been made to FRAP procedure since it was first introduced, the fundamental idea has not changed (Lippincott-Schwartz et al. 2018).

In typical FRAP experiments, a short sequence of images is acquired prior to photobleaching. These serve to document the initial spatial distribution of the fluorescent molecule. The next step is photobleaching: a small defined region of interest is briefly illuminated with high intensity light, usually delivered by a laser. This triggers an irreversible change in the chemistry of the fluorophore (typically GFP) which causes a permanent loss in fluorescent properties. This creates a high concentration of photobleached (or simply bleached) protein molecules within the region of interest. Next, the laser intensity is attenuated in order to acquire a longer sequence of images, ideally with minimal photobleaching. During this period the motion of both non-bleached and bleached GFP molecules will lead to the spatial re-distribution of the fluorescent signal. Passive transport processes, such as Brownian motion, will create a net transfer of bleached molecules out of (and a net transfer of unbleached molecules into) the region of interest, causing the cell to relax towards equilibrium. This is referred to as the fluorescence recovery. The average intensity of fluorescent emission from the region of interest is recorded against time to construct the fluorescence recovery curve (White and Stelzer 1999; Meyvis et al. 1999; Reits and Neefjes 2001; Carrero et al. 2003).

The earliest FRAP experiments (conventional FRAP) were conducted using a static laser that was attenuated by placing neutral density filters in front of the beam (Jacobson et al. 1976). As fluorescence microscopy proliferated during the 1980s, the confocal scanning laser microscope (CSLM) was developed (Amos and White 2003). This type of microscopy relies on raster scanning a laser beam over an area of interest. Modern FRAP apparatus essentially consists of a CSLM and an acousto-optical modulator which is capable of rapidly varying the intensity of the CSLM laser as it scans across the sample. During image acquisition a low laser intensity is used on the whole field of view, whereas during photobleaching the laser intensity is increased, and the scan area restricted to just the region that is being targeted for bleaching. A static beam can only yield a fluorescence recovery curve, but with modern confocal scanning FRAP almost any desired pattern can be bleached into fluorescent samples at high definition and then imaged (Wedekind et al. 1994).

### Quantitative analysis of fluorescence recovery after photobleaching

Quantitative analysis of FRAP data is made possible by mathematical modelling. Many different models have been brought forward, beginning in the 1970s with relatively simple analytical models, based on partial differential equations that are solved (typically under idealised conditions) in order to derive an expression for the recovery curve. By fitting a model to experimental data, estimates of the model parameters are produced. The earliest FRAP models [the first being published in Axelrod et al. (1976)] were single species analytic models of protein transport within cellular membranes, due to diffusion or electrophoresis (Axelrod et al. 1976; Soumpasis 1983). Many different models have since been proposed, including simplified one-dimensional models (Ellenberg et al. 1997; Houtsmuller et al. 1999) and more complicated three-dimensional models (Braeckmans et al. 2003; Braga et al. 2004; Mazza et al. 2008).

The principal disadvantage of analytical modelling is that it is almost always necessary to make simplifying assumptions, for example that the system is homogeneous, that the system is infinitely large or that photobleaching is effectively instantaneous. The latter assumption is a problem in confocal scanning FRAP, since photobleaching requires repeated scanning of the region of interest which typically takes several seconds (Kang et al. 2009). This has forced analytical modellers to make phenomenological assumptions about the distribution of fluorescent material immediately after photobleaching (Braga et al. 2007; Kang et al. 2009, 2010). There also exists a variety of computational models that need not make any of these simplifying assumptions, of which there are two main types: continuum models in which a partial differential equation is solved numerically (Beaudouin et al. 2006; Blumenthal et al. 2015; Moraru et al. 2008; Bläßle et al. 2018; Röding et al. 2019), and stochastic approaches that track the diffusion and interactions of individual molecules (Nicolau et al. 2007; Vilaseca et al. 2011; Groeneweg et al. 2014). Computational models have the clear advantage over analytical models that they may incorporate greater complexity yet have the disadvantage that they may be time-consuming to run (particularly Monte Carlo methods).

One of the most significant developments in the history of FRAP modelling was the introduction of models that incorporate binding kinetics, either to immobile interacting partners within the cell or to partners with different diffusion properties (Kaufman and Jain 1990; Carrero et al. 2003; Sprague et al. 2004; Lin and Othmer 2017; Hinow et al. 2006; Phair et al. 2004; Kang et al. 2010; Braga et al. 2007). Knowledge of kinetic properties may yield important biological conclusions about how proteins function (Mueller et al. 2010). To give an example, Ege *et al.* established quantitative differences in molecular association and dissociation rates of a regulatory protein, YAP1, as evidence of qualitative biological differences between the normal and cancer-associated variants of fibroblasts (Ege et al. 2018).

While there is much value in FRAP mathematical modelling, various problems remain. First, FRAP studies using different kinetic models have been shown to arrive at very different predictions for the same or similar proteins due to technical issues (rather than genuine biological differences) (Mueller et al. 2010). Secondly, fits to FRAP data are not necessarily unique, which diminishes their usefulness (Sadegh Zadeh et al. 2006). In this article, we will seek to provide additional clarity by deriving mathematical conditions, in terms of model parameters and experimental parameters (such as recording frame rate), which guarantee that all model parameters are theoretically estimable from FRAP data. When this is the case, we will say that the model is tractable.

In Sect. 2 we will introduce the two-species reaction diffusion model that we will use throughout. In Sect. 3 we will present new analytic FRAP formulae and formally re-derive existing ones using asymptotic methods (derivations may be found in appendix A). Invoking the concept of Fisher information, we will infer sufficient conditions to ensure FRAP model tractability. In Sect. 4 we present the computational methods used to test our theoretical predictions from Sect. 3. Further numerical investigation will inform as to the best course of action in cases where the tractability conditions do not hold. Computational results are discussed in Sect. 5. In Sect. 6 we propose a general method to determine when full parameter fitting is possible and when extra measures will be required. Finally, possibilities for future work considered in Sect. 7.

## Mathematical model

We assume that a diffusible protein species, A, associates reversibly with a homogeneously distributed binding partners, B, to form a complex molecules, C. We also assume that the number of molecules involved is large enough for the law of mass action to be applicable so that, in a well-mixed system, the concentrations of A, B and C evolve according to,

1 $$\begin{aligned} {\left\{ \begin{array}{ll} \frac{\text {d}}{\text {d}t}\mathrm {[A]}= -\bar{k}_\mathrm {on} \mathrm {[A]}\mathrm {[B]}+ k_\mathrm {off}\mathrm {[C]},\\ \frac{\text {d}}{\text {d}t}\mathrm {[B]}= -\bar{k}_\mathrm {on} \mathrm {[A]}\mathrm {[B]}+ k_\mathrm {off}\mathrm {[C]},\\ \frac{\text {d}}{\text {d}t}\mathrm {[C]}= \bar{k}_\mathrm {on} \mathrm {[A]}\mathrm {[B]}- k_\mathrm {off}\mathrm {[C]}, \end{array}\right. } \end{aligned}$$

where $\mathrm {[X]}$ denotes the concentration of X.

Prior to the FRAP experiment, protein A is tagged with a fluorescent probe. We also assume that system (1) reaches chemical equilibrium before the experiment begins. Let $u(\mathbf {x},t)$ be the concentration of species A at point $\mathbf {x}$ and time *t* that is fluorescent (not photobleached), and $D_u$ be the diffusivity of A (note that photobleaching is assumed not to alter the diffusivity or reactivity of the molecules). Likewise, let $v(\mathbf {x},t)$ be the concentration of the fluorescent C species and $D_v$ its diffusivity. Similarly, let the concentrations of photobleached A and C be $\bar{u}$ and $\bar{v}$ respectively.

**Fig. 1:**
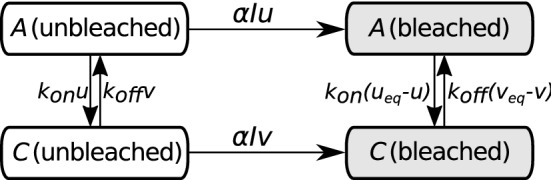
Schematic representation of model (3). Arrows, which indicate chemical reactions and photobleaching, are labelled with the reaction rates derived from the principle of mass action. Note that the bleached and unbleached concentrations of A and C sum to $u_\mathrm {eq}$ and $v_\mathrm {eq}$ respectively, since photobleaching does not perturb the overall chemical equilibrium, only the fluorescence equilibrium

As A is the tagged species, only molecules of A, or molecules which contain A, may be fluorescent. Hence a molecule of C is fluorescent only if it contains a fluorescent A, as B is not tagged. Association of fluorescent A with B will always form fluorescent C, and dissociation of fluorescent C will always release fluorescent A. Both A and C can be photobleached by exposure to high intensity light, which we assume has intensity $I(\mathbf {x},t)$ at position $\mathbf {x}$ and time *t*. Making the simplifying assumption (Lorén et al. 2015) that photobleaching is a first order process, the rate of bleaching per unit concentration is $\alpha I(\mathbf {x},t)$, where $\alpha$ is the sensitivity of the fluorescent probe to photobleaching. The resulting system of equations is,

2 $$\begin{aligned} {\left\{ \begin{array}{ll} \frac{\partial u}{\partial t}(\mathbf {x},t) = -\bar{k}_\mathrm {on} [B] u(\mathbf {x},t) + k_\mathrm {off}v(\mathbf {x},t) +D_u\nabla ^2u(\mathbf {x},t) - \alpha I(\mathbf {x},t)u(\mathbf {x},t), \\ \frac{\partial v}{\partial t}(\mathbf {x},t) = \bar{k}_\mathrm {on} [B] u(\mathbf {x},t) - k_\mathrm {off}v(\mathbf {x},t) +D_v\nabla ^2v(\mathbf {x},t) - \alpha I(\mathbf {x},t)v(\mathbf {x},t),\\ \frac{\partial \bar{u}}{\partial t}(\mathbf {x},t) = -\bar{k}_\mathrm {on} [B] \bar{u}(\mathbf {x},t) + k_\mathrm {off}\bar{v}(\mathbf {x},t) +D_u\nabla ^2\bar{u}(\mathbf {x},t) + \alpha I(\mathbf {x},t)u(\mathbf {x},t), \\ \frac{\partial \bar{v}}{\partial t}(\mathbf {x},t) = \bar{k}_\mathrm {on} [B] \bar{u}(\mathbf {x},t) - k_\mathrm {off}\bar{v}(\mathbf {x},t) +D_v\nabla ^2\bar{v}(\mathbf {x},t) + \alpha I(\mathbf {x},t)v(\mathbf {x},t), \end{array}\right. } \end{aligned}$$

(also see Fig. 1 for a schematic representation).

It is clear by conservation of mass that $u + \bar{u} = u_\mathrm {eq}$, a constant (likewise $v + \bar{v} = v_\mathrm {eq}$). Note that $u_\mathrm {eq}$ and $v_\mathrm {eq}$ are the pre-bleach equilibrium concentrations of fluorescent A and C respectively, assuming that all material is fluorescent prior to photobleaching. Using the fact that $\bar{u} = u_\mathrm {eq}- u$ and $\bar{v} = v_\mathrm {eq}-v$, (2) simplifies to

3 $$\begin{aligned} {\left\{ \begin{array}{ll} \frac{\partial u}{\partial t}(\mathbf {x},t) = -k_\mathrm {on}u(\mathbf {x},t) + k_\mathrm {off}v(\mathbf {x},t) +D_u\nabla ^2u(\mathbf {x},t) - \alpha I(\mathbf {x},t)u(\mathbf {x},t), \\ \frac{\partial v}{\partial t}(\mathbf {x},t) = +k_\mathrm {on}u(\mathbf {x},t) - k_\mathrm {off}v(\mathbf {x},t) +D_v\nabla ^2v(\mathbf {x},t) - \alpha I(\mathbf {x},t)v(\mathbf {x},t), \end{array}\right. } \end{aligned}$$

where $k_\mathrm {on}= \bar{k}_\mathrm {on}\mathrm {[B]}$, which is a constant as the concentration of binding sites is not altered by photobleaching. Model (3) has appeared previously in several quantitative FRAP studies, some of which assume the immobility of the binding sites (*i.e.* $D_v=0$) (Kaufman and Jain 1990; Sprague et al. 2004; Hinow et al. 2006; Beaudouin et al. 2006; Mueller et al. 2008; Tsibidis 2009), while others allow for the possibility of mobile sites ($D_v>0$) (Braga et al. 2007; Berkovich et al. 2011; Montero Llopis et al. 2012) (this study of Ras by Kang et al. (2010) is an empirical example of a molecule with a non-zero diffusivity in the bound state).

For convenience we measure fluorescence in units such that the total pre-bleach fluorescence is 1 ($u_\mathrm {eq}+v_\mathrm {eq}=1$), which implies that

4 $$\begin{aligned} u_\mathrm {eq}=\frac{k_\mathrm {off}}{k_\mathrm {on}+k_\mathrm {off}}, \quad v_\mathrm {eq}=\frac{k_\mathrm {on}}{k_\mathrm {on}+k_\mathrm {off}}. \end{aligned}$$

Model (3) includes four physical parameters, the diffusivities $D_u$ and $D_v$ and the reaction rates $k_\mathrm {on}$ and $k_\mathrm {off}$. In what follows we will seek to determine the reliability with which these four parameters (or combinations thereof) can be measured experimentally by fitting (3) to simulated synthetic data.

We assume the system (3) to be radially symmetric. Let $r=\sqrt{x^2+y^2}$, and non-dimensionalise by setting $r'=r/r_n$, where $r_n$ is the characteristic radius of the bleach region of interest and $t'=k_\mathrm {on}t$. The resulting equations (given negligible laser intensity) are

5 $$\begin{aligned} {\left\{ \begin{array}{ll} \frac{\partial u}{\partial t'} = -u + \kappa v + \eta \frac{1}{r'}\frac{\partial }{\partial r'}\left( r'\frac{\partial u}{\partial r'} \right) , \\ \frac{\partial v}{\partial t'} = u - \kappa v + \delta \eta \frac{1}{r'}\frac{\partial }{\partial r'}\left( r'\frac{\partial v}{\partial r'} \right) , \end{array}\right. } \end{aligned}$$

where

6 $$\begin{aligned} \delta =D_v/D_u, \quad \kappa = k_\mathrm {off}/k_\mathrm {on}, \quad \eta =D_u /(k_\mathrm {on}r_n^2) \end{aligned}$$

are positive dimensionless parameters. We will consider the initial value problem for (5) on an infinite spatial domain with far field conditions

7 $$\begin{aligned} \lim _{r'\rightarrow \infty } u(r',t')=u_\mathrm {eq}, \quad \lim _{r'\rightarrow \infty } v(r',t')=v_\mathrm {eq}, \end{aligned}$$

and initial conditions

8 $$\begin{aligned} u(r',0)=U_0(r')=u_\mathrm {eq}H(r'-1), \quad v(r,0)=V_0(r')=v_\mathrm {eq}H(r'-1), \end{aligned}$$

where *H* is the Heaviside step function. Initial conditions (8) are appropriate if all available material inside the region of interest is bleached instantaneously. We will show that the orders of magnitude of the dimensionless parameters $\eta$, $\kappa$ and $\delta$ control the identifiability of the model parameters, $D_u$, $D_v$, $k_\mathrm {on}$ and $k_\mathrm {off}$.

## Inverse modelling problem

The inverse modelling problem is the problem of minimising an appropriate objective function in order to obtain a maximum likelihood estimate for the values of the model parameters. If all model parameters are identifiable given some data, we will refer to the inverse modelling problem as tractable.

In keeping with numerous prior studies (Axelrod et al. 1976; Soumpasis 1983; Sprague et al. 2004; Kang et al. 2009) we define the fluorescence recovery curve as the average light intensity of fluorescent emission across the region of interest (ROI),

9 $$\begin{aligned} F(t) = \frac{\iint _\text {ROI} w(r,t) \text {d}A}{\iint _\text {ROI} \text {d}A}, \end{aligned}$$

where

10 $$\begin{aligned} w(r,t)= u(r,t)+v(r,t). \end{aligned}$$

To be consistent with the initial conditions (8), the region of interest, which is the area covered by the laser, is assumed to be a circular region of radius $r_n$. Although *F*(*t*) in (9) is a continuous function of time, in practice only finitely many data points $F(t_i)$ may be acquired at discrete times, $t_i$. Let $\Delta t = t_{i+1}-t_i$ for all *i* be the time step, so that $1/\Delta t$ is the frame rate of the imaging process; let $Y_i$ be the fluorescence recovery curve data values at the sample times $t_i$; and $F_i({\varvec{\theta }})=F(t_i;\theta )$, with $\theta$ being the vector of model parameters. We assume that empirical data may be described by the sum of the output of the mathematical model and a stochastic variable such that,

11 $$\begin{aligned} Y_i = F_i({\varvec{\theta }}) + \sigma _i \xi _i, \end{aligned}$$

where the $\xi _i$ are normally distributed random variables and the $\sigma _i$ account for the scale of the observational uncertainty. This assumption is appropriate if the the model accurately captures the underlying dynamics of the system under investigation and experimental errors are normally distributed. The objective function may be defined as follows

12 $$\begin{aligned} \phi (\theta ) = \sum _i \frac{(F_i(\theta ) - Y_i)^2}{2 \sigma _i^2}, \end{aligned}$$

where the factor of 2 is purely for notational convenience. The global minimum of the objective function, $\theta ^*$, corresponds to a maximum likelihood estimate of model parameters (White et al. 2016). The identifiability of the model parameters is given by the Fisher Information Matrix (FIM) (Rao 1992; Akaike 1998), which in this case is the Hessian matrix of the objective function,

13 $$\begin{aligned} I_{\mu \nu }= \left. \frac{\partial ^2 \phi }{\partial \theta _\mu \partial \theta _\nu } \right| _{\theta ^*}, \end{aligned}$$

which gives

$$\begin{aligned} I_{\mu \nu }= \sum _i \frac{1}{\sigma _i^2} \frac{\partial }{\partial \theta _\mu }\left( F_i\frac{\partial F_i}{\partial \theta _\nu } - Y_i\frac{\partial F_i}{\partial \theta _\nu } \right) = \sum _i \frac{1}{\sigma _i^2}\left( \frac{\partial F_i}{\partial \theta _\mu }\frac{\partial F_i}{\partial \theta _\nu } +\frac{\partial ^2 F_i}{\partial \theta _\nu \partial \theta _\mu } (F_i - Y_i) \right) . \end{aligned}$$

If the system is well-described by the model, then $F_i(\theta ^*)=Y_i$, so the term involving second derivatives vanishes and the FIM simplifies to

14 $$\begin{aligned} I_{\mu \nu }= \sum _i \frac{1}{\sigma _i^2} \left. \frac{\partial F_i}{\partial \theta _\mu }\frac{\partial F_i}{\partial \theta _\nu }\right| _{\theta ^*}. \end{aligned}$$

This relation is exact in our case since our data are synthetic. If an eigenvector of the FIM has a large corresponding eigenvalue, then the combination of parameters given by the eigenvector is identifiable (Gutenkunst et al. 2007). Models containing a mixture of identifiable and non-identifiable parameters (for which the eigenvalues of the FIM are spread across a logarithmic scale) are said to be sloppy (White et al. 2016). Sloppy models characteristically include certain parameters, or combinations of parameters, in which even substantial variations do not significantly affect the behaviour of the dependent variables. In geometric terms, there is a manifold within the space of model parameters which is a flat minimum of the objective function so that the global minimum cannot be easily located. Numerous sloppy models have been identified within the mathematical biology literature, usually those with large numbers of parameters (Gutenkunst et al. 2007; Daniels et al. 2008; Machta et al. 2013; Transtrum et al. 2015). Even though the FRAP model (3) has only four parameters, we will show that under certain circumstances it may be sloppy in the sense of having a mixture of identifiable and unidentifiable parameters.

### Asymptotic approximations

In this section, we will handle important special cases of (5) analytically under idealised circumstances (*i.e.* subject to far-field conditions (7) and initial conditions (8), paying special attention to the effect of varying $\eta$ and $\kappa$ on parameter identifiability. It is useful at this stage to introduce the following function, which is the recovery curve of a radially symmetric, single species, pure diffusion FRAP model with Heaviside step function initial conditions (Soumpasis 1983):

15 $$\begin{aligned} \mathscr {F}_S (z) = e^{-z}(I_0(z) + I_1(z)) = \sqrt{\frac{2}{\pi z}}\left( 1- \frac{1}{8z} + ... \right) , \end{aligned}$$

where $I_0$ and $I_1$ are modified Bessel functions of the first kind. The series expression is due to the asymptotic expansion of Abramowitz and Stegun (1972).

#### Rapid equilibration ($\eta \gg 1$)

In the limit as $\eta \rightarrow \infty$, the dynamics of the fluorescence recovery arising from system (5) subject to (7) and (8) admit a small parameter $\varepsilon =1/\eta \ll 1$. The recovery curve is then well approximated (see appendix A.1) by

16 $$\begin{aligned} F(t)=\frac{k_\mathrm {off}}{k_\mathrm {on}+k_\mathrm {off}}\mathscr {F}_S\left( \frac{r_n^2}{2 D_u t}\right) + \frac{k_\mathrm {on}}{k_\mathrm {on}+k_\mathrm {off}} \left( 1 - e^{-k_\mathrm {off}t}\left[ 1 - \mathscr {F}_S\left( \frac{r_n^2}{2 D_v t}\right) \right] \right) , \end{aligned}$$

which holds for $\delta =O(\varepsilon )$, where $\delta =D_v/D_u$.

**Fig. 2:**
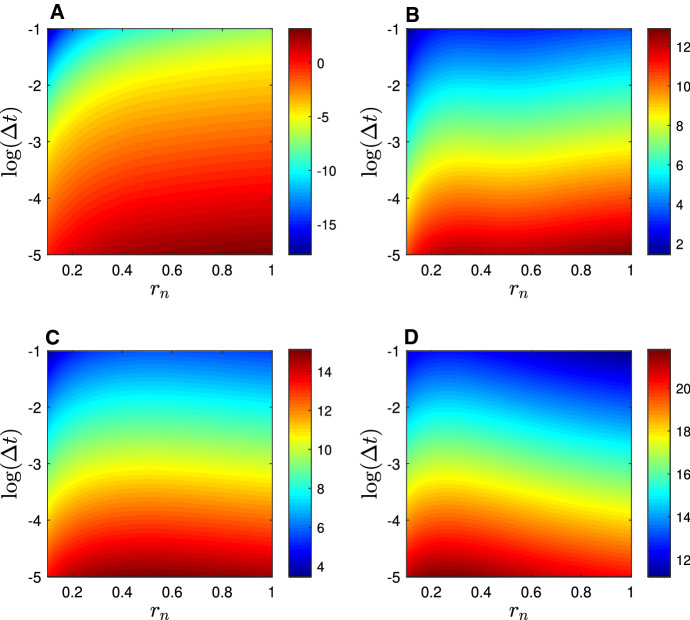
Logarithm of the eigenvalues, $\log (\lambda _i)$, of the Fisher information matrix computed from formula (16) for different values of the $r_n$ and $\Delta t$. Each subplot corresponds to one of the four eigenvalues, **a** $\lambda _1$, **b** $\lambda _2$, **c** $\lambda _3$ and **d** $\lambda _4$

**Fig. 3:**
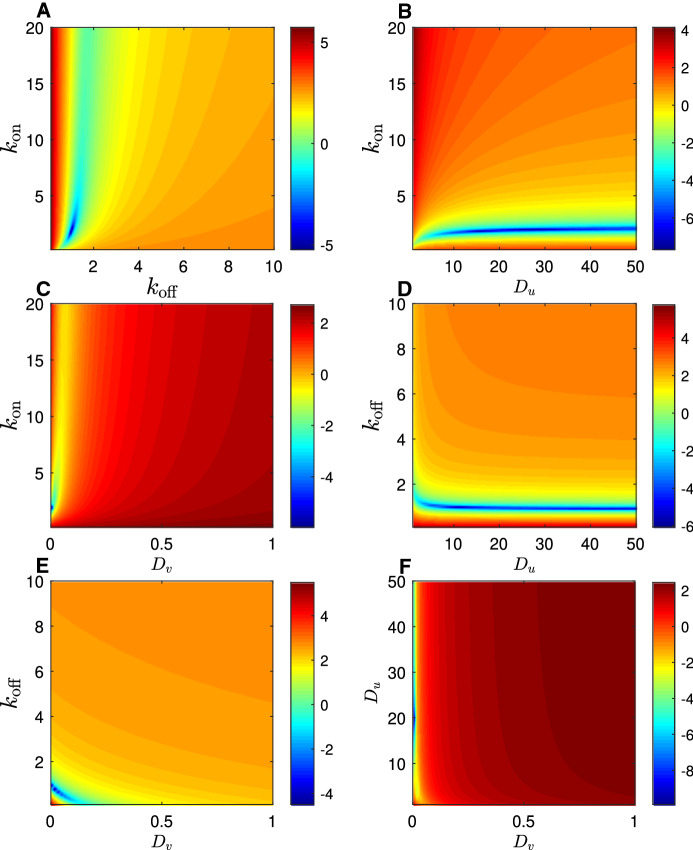
Logarithm of the numerically constructed objective function, $\log (\phi )$, in the rapid equilibration (reaction limited) regime. Colour indicates the size of the sum of square errors between a single simulated fluorescence recovery curve spanning 15 seconds (1024 data points) generated with parameter values $D_u=20.0 \mu m^2 s^{-1}, D_v = 0.00 \mu m^2 s^{-1}, k_\mathrm {on}=2.00s^{-1}, k_\mathrm {off}=1.00s^{-1}$, and a secondary simulated recovery curve generated with indicated parameter values. Each subplot displays variation in one of the six possible pairs of parameters, with the remaining two parameters held at the correct value in each case

From expansion (15) it is clear that $\lim _{z\rightarrow 0}\mathscr {F}_S(z)=1$ and that $\lim _{z\rightarrow \infty }\mathscr {F}_S(z)=0$, so, if the slow diffusion time scale is sufficiently long ($r_n^2/D_v\gg 1$) and the time scale of rapid diffusion is sufficiently short ($r_n^2/D_u\ll 1$), then for all $t=O(1)$ (16) reduces to the well known formula (Bulinski et al. 2001; Dundr et al. 2002; Phair et al. 2004; Rabut et al. 2004; Sprague et al. 2004) for ‘reaction limited’ dynamics,

17 $$\begin{aligned} F(t)= \frac{k_\mathrm {off}}{k_\mathrm {on}+k_\mathrm {off}} + \frac{k_\mathrm {on}}{k_\mathrm {on}+k_\mathrm {off}} \left( 1 - e^{-k_\mathrm {off}t}\right) = 1-\frac{k_\mathrm {on}}{k_\mathrm {on}+k_\mathrm {off}} e^{-k_\mathrm {off}t}, \end{aligned}$$

or equivalently,

18 $$\begin{aligned} F(t)=1 - v_\mathrm {eq}e^{-k_\mathrm {off}t}. \end{aligned}$$

Formula (18) suggests *prima facie* that the dissociation rate $k_\mathrm {off}$ is the only measurable model parameter, yet this is not necessarily true. The eigenvalues of the Fisher information matrix derived from formula 16 are plotted in Fig. 2. One of the eigenvalues (Fig. 2a) is many orders of magnitude smaller than the other three. On this basis we expect that there will be a manifold within the parameter space which represents a flat minimum of the objective function. This is quite clearly visible in Fig. 3b, d and f, showing that the diffusivity, $D_u$, is inestimable. The fluorescence recovery in this case is bi-phasic; there is an early diffusion-dominated phase which occurs imperceptibly quickly unless the time step, $\Delta t$, is much smaller than the time scale of diffusion across the bleach region of interest. Equivalently, $D_u$ is only estimable if

19 $$\begin{aligned} \Delta t \ll \frac{r_n^2}{D_u}. \end{aligned}$$

Returning briefly to Fig. 2a, it is quite clear that the magnitude of the smallest eigenvalue is increased as $\Delta t$ decreases and $r_n$ increases. Interestingly, two other eigenvalues (Fig. 2c, d) visibly decline, for fixed $\Delta t$, as $r_n$ increases. Notwithstanding, it is clear that every model parameter is estimable in this case provided condition (19) holds.

#### Intermediate equilibration ($\eta =O(1)$)

No known approximations describe the dynamics of the intermediate case in which $\eta = O(1)$, but this does not mean that it is impossible to analyse. In order to derive formula (17) we introduced an asymptotic expansion in terms of a small parameter $\varepsilon =1/\eta$ to produce an approximation which holds whenever $\eta$ is large. By extending our asymptotics to include first order terms (see appendix A.2), we are able to produce an approximation which is accurate for somewhat smaller values of $\eta$ and so gives some insight into the behaviour of the system as it approaches $\eta = O(1)$. The first-order extension of formula (17) is

20 $$\begin{aligned} F(t) = 1-\frac{k_\mathrm {on}}{k_\mathrm {on}+ k_\mathrm {off}} e^{-k_\mathrm {off}t} -\frac{k_\mathrm {on}r_n}{3 D_u} e^{-k_\mathrm {off}t}\left( \frac{k_\mathrm {off}+ k_\mathrm {on}k_\mathrm {off}t}{k_\mathrm {on}+k_\mathrm {off}}\right) , \end{aligned}$$

which holds for $r_n^2/D_v\ll 1$ and $t\gg \varepsilon$. In contrast with formula (18), $D_u$ appears explicitly in (20), which implies that it could be estimated if the other model parameters were known. The full significance of this result will be discussed in Sect. 3.3.

**Fig. 4:**
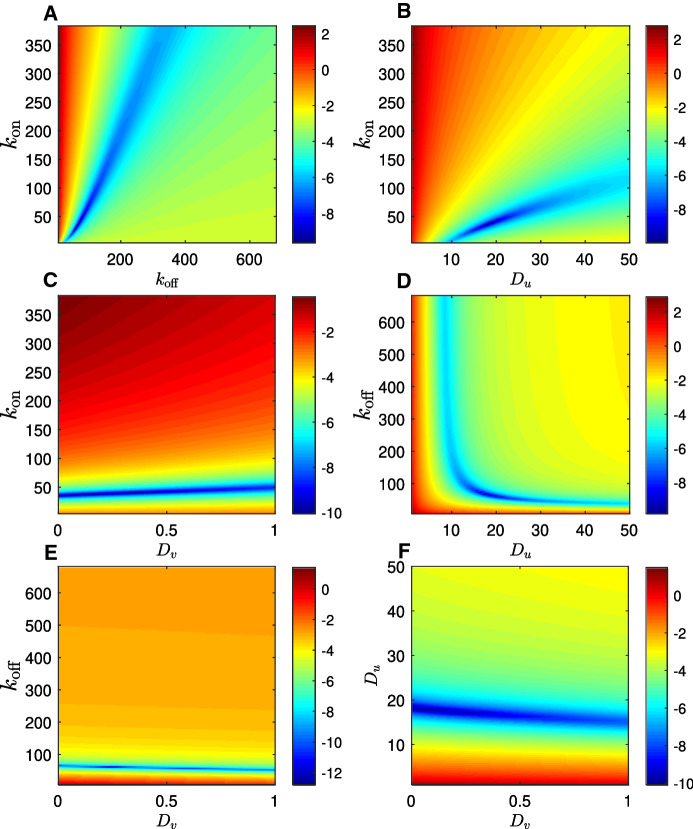
Logarithm of the numerically constructed objective function, $\log (\phi )$, in the intermediate equilibration regime. Colour indicates the size of the sum of square errors between a single simulated fluorescence recovery curve spanning 15 seconds (1024 data points) generated with parameter values $D_u=18.1 \mu m^2 s^{-1}, D_v = 0.0718 \mu m^2 s^{-1}, k_\mathrm {on}=38.3s^{-1}, k_\mathrm {off}=68.2$, and a secondary simulated recovery curve generated with indicated parameter values. Each subplot displays variation in one of the six possible pairs of parameters, with the remaining two parameters held at the correct value in each case

By contrasting Figs. 3 and 4, it can be seen quite clearly how reducing the value of the dimensionless parameter $\eta$ changes the shape of the objective function. In particular, in the subplots that involve $D_u$ (Fig. 4b, d, f), there is a clear unique local minimum of the objective function (in contrast with the manifold in Fig. 3b, d, f) which tends to support the prediction that $D_u$ is estimable when $\eta =O(1)$ (at least when other parameters are known).

**Fig. 5:**
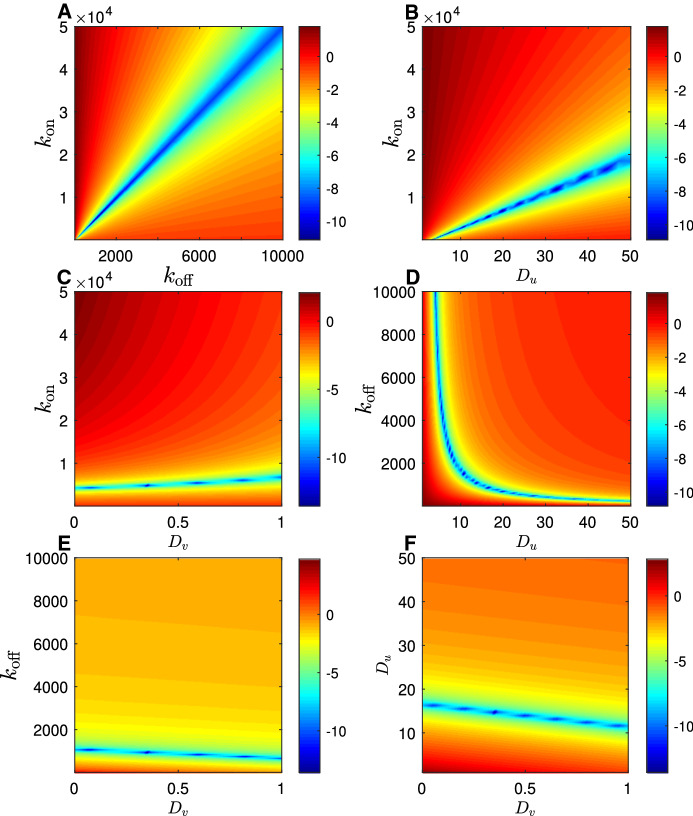
Logarithm of the numerically constructed objective function, $\log (\phi )$, in the slow equilibration (effective diffusion) regime. Colour indicates the size of the sum of square errors between a single simulated fluorescence recovery curve spanning 15 seconds (1024 data points) generated with parameter values $D_u=15.0 \mu m^2 s^{-1}, D_v = 0.374 \mu m^2 s^{-1}, k_\mathrm {on}=1000s^{-1}, k_\mathrm {off}=5000$, and a secondary simulated recovery curve generated with indicated parameter values. Each subplot displays variation in one of the six possible pairs of parameters, with the remaining two parameters held at the correct value in each case

#### Slow equilibration ($\eta \ll 1$)

In the limit $\eta \rightarrow 0$, we define the small parameter by $\varepsilon =\eta$. The fluorescence recovery approximation is simply

21 $$\begin{aligned} F(t) = \mathscr {F}_S \left( \frac{r_n^2}{2D_\text {eff} t} \right) , \end{aligned}$$

where $D_\text {eff}$, a straightforward generalisation of the effective diffusivity defined by Crank (1975), is

22 $$\begin{aligned} D_\text {eff} = \frac{k_\mathrm {on}D_v + k_\mathrm {off}D_u}{k_\mathrm {off}+ k_\mathrm {on}}. \end{aligned}$$

As the recovery curve (21) depends only on $D_\mathrm {eff}$, this is the only estimable combination of parameters. As $\eta \rightarrow 0$ , a manifold within the parameter space, defined by (22), emerges upon which the value of the objective function is approximately zero. In Figure 5, which is clearly visible in any subplot of Fig. 5. No pair of parameters could be estimated even if the values of the other two were known. It would be necessary to determine three of the parameters to determine the fourth. If the diffusivities, $D_u$ and $D_v$, could be independently determined, then at most the ratio of the reaction rates, $\kappa = k_\mathrm {off}/k_\mathrm {on}$ could be estimated.

Like the rapid equilibration case, the slow equilibration recovery is bi-phasic. The early phase consists of a rapid convergence to local chemical equilibrium between the bound and unbound species (*v* and *u* respectively) which is imperceptible because it does not alter the total concentration, *w*.

#### Asymmetric reaction rates ($\kappa \gg 1$)

If we take $\kappa \rightarrow \infty$, we find that $u_\mathrm {eq}=\kappa /(1+\kappa )\rightarrow 1$, so almost all available material will be in the unbound state, and the system will be closely approximated by pure diffusion. The recovery curve is

23 $$\begin{aligned} F(t) = \mathscr {F}_S \left( \frac{r_n^2}{2D_u t} \right) . \end{aligned}$$

Since,

24 $$\begin{aligned} D_\text {eff} = \frac{D_v + \kappa D_u}{\kappa + 1}, \end{aligned}$$

it is clear that $D_\text {eff}\rightarrow D_u$ as $\kappa \rightarrow \infty$. In effect, the $\kappa \gg 1$ case coincides with the $\eta \ll 1$ case, except that $\kappa =k_\mathrm {off}/k_\mathrm {on}$ could not be estimated in the $\kappa \gg 1$ case even if $D_u$ and $D_v$ could be independently measured.

#### Asymmetric reaction rates ($\kappa \ll 1$)

As $\kappa \rightarrow 0$ almost all available molecules are in a bound state, such that the recovery curve can be approximated by

25 $$\begin{aligned} F(t) = \mathscr {F}_S \left( \frac{r_n^2}{2D_v t} \right) . \end{aligned}$$

and $D_v$ is the only measurable parameter. As $\kappa \rightarrow 0$, $D_\text {eff}\rightarrow D_v$, so this case coincides with the $\eta \ll 1$ case, except that $\kappa$ itself is always inestimable.

### Parameter identifiability

Here we will summarise the conditions which guarantee parameter identifiability in FRAP modelling. Suppose we have a theoretical recovery curve based on the solution to a mathematical model $F(t;\theta )$ for parameter values $\theta$, and some recovery curve data $F_\text {Data}(t)$. We can define the objective function, $\phi (\theta )$, to be the residual sum of squared errors (without the scaling with $\sigma _i$ used in (12)),

26 $$\begin{aligned} \phi (\theta ) = \sum _{i} (F(t_i;\theta )-F_\text {Data}(t_i))^2. \end{aligned}$$

We have four physical model parameters that are unknown, $\theta = (D_u,D_v,k_\mathrm {on},k_\mathrm {off})$ and two experimental parameters: $r_n$, the radius of the bleach region of interest and $\Delta t=t_{i+1}-t_{i}$ the time interval between data points.

Of the cases we have considered in Sect. 3.1, the inverse modelling problem was tractable only in the rapid equilibration ($\eta \gg 1$) when condition (19) holds. This means that we require the following conditions on the physical parameters

27 $$\begin{aligned} \frac{k_\mathrm {off}}{k_\mathrm {on}} =O(1), \quad D_v \ll D_u, \end{aligned}$$

and the following conditions on the experimental parameters

28 $$\begin{aligned} \frac{D_u}{k_\mathrm {on}r_n^2}\ll 1, \Delta t \ll \frac{r_n^2}{D_u}. \end{aligned}$$

These results imply that smaller bleach region radius and higher frame rate data acquisition are generally preferable in principle. However, this is not necessarily practical; $r_n$ cannot be reduced arbitrarily as the resolution of an optical system is limited by diffraction. Although conditions (27) and (28) appear quite specific, we expect that systems in which they are satisfied will be relatively common. For example, many different nuclear proteins have been found to have a high mobility (van Royen et al. 2009; Phair and Misteli 2000). Highly mobile proteins such as those found within the cell nucleus will satisfy condition (27) except in extreme cases of highly transient binding interactions.

### Confocal scanning FRAP

As we discussed in Sect. 1, confocal scanning FRAP, unlike conventional FRAP, may yield a detailed recording of an entire cell. In this case, we may attempt to fit the total fluorescence $w(\mathbf {x},t)$, not just the recovery curve *F*(*t*). Under the assumption of radial symmetry, let

29 $$\begin{aligned} w(r,t;\theta )=u(r,t;\theta )+v(r,t;\theta ), \end{aligned}$$

for some parameter values, $\theta$, and let $w_\mathrm {Data}(r,t)$ be some appropriate fluorescence microscopy data. The objective function in this case is defined as

30 $$\begin{aligned} \phi _\text {Space} (\theta )= \sum _{i} \sum _{j} (w_\text {Data}(r_j, t_i) - w(r_j, t_i))^2. \end{aligned}$$

It has already been observed that the process of averaging across the bleach region of interest to compute the recovery curve effectively destroys a significant amount of information (Orlova et al. 2011; Seiffert and Oppermann 2005), so we expect that it will be advantageous to define the objective function as in (30). Here we will derive simple conditions to ensure parameter estimability in confocal scanning FRAP.

Once again, we have four physical parameters, $\theta = (D_u,D_v,k_\mathrm {on},k_\mathrm {off})$, though this time we have three experimental parameters: $\Delta r$, the length scale of a pixel of the micrograph; $\Delta t$, the duration of one frame; and *L*, the length scale of the whole field of view.

We could, in principle, construct a recovery curve of radius $r_n$ so that $\eta =D_u/(k_\mathrm {on}r_n)\gg 1$, provided that $\Delta r < r_n$ (clearly we cannot have a recovery curve radius smaller than one pixel). As we saw in Sect. 3.1.1, we could use this recovery curve to estimate $k_\mathrm {on}$, $k_\mathrm {off}$ and $D_v$, but not necessarily $D_u$ except for very high frame rate data. Likewise, we could construct a second recovery curve of radius $r_n'$ so that $\eta '=D_u/(k_\mathrm {on}r_n')=O(1)$ provided that $L > r_n'$. From the results of Sect. 3.1.2 (formula (20)) we know that $D_u$ will be estimable if $\eta ' = O(1)$ or greater, as long as the other model parameters are known, but this is certainly the case because estimates can be obtained from the first recovery curve of radius $r_n$. Moreover, there is no theoretical reason to suppose that the two recovery curves would actually be necessary, as the objective function (30) contains information about the redistributive dynamics of the system under investigation on all length scales between $\Delta r$ and *L*. In summation, we expect that the inverse modelling problem of confocal scanning FRAP will be fully tractable as long as

31 $$\begin{aligned} \frac{k_\mathrm {off}}{k_\mathrm {on}} =O(1), \quad D_v \ll D_u, \end{aligned}$$

and

32 $$\begin{aligned} \frac{D_u}{k_\mathrm {on}\Delta r^2}\gg 1, \quad \frac{D_u}{k_\mathrm {on}L^2} \lesssim 1. \end{aligned}$$

There is also an extremely weak implicit constraint on $\Delta t$, that the frame rate is not so low that the fluorescence recovery is totally imperceptible.

## Computational methodology

The analysis in Sect. 3 has two limitations. First, it is local to the optimal point and does not reveal anything about the viability of global parameter fitting with general initial guesses that may be far from the global minimum. Secondly, it applies only to the idealised case with step function initial conditions. In this section, we will introduce the computational methods by which we aim to test our theoretical predictions from Sect. 3 and extend our results to the global parameter fitting problem with non-ideal initial conditions.

We simulate the FRAP model (3) numerically with the laser profile *I*(*r*, *t*) being given in terms of the Heaviside step function as

33 $$\begin{aligned} I(r,t) = H(r_n-r)H(t_\mathrm {bleach}-t). \end{aligned}$$

We impose zero-flux boundary conditions on a disk34and likewise for *v*. The radially symmetric Laplacian is

35 $$\begin{aligned} \nabla ^2u = {\left\{ \begin{array}{ll} 2\frac{\partial ^2 u}{\partial r^2}, \qquad \qquad r=0,\\ \frac{1}{r}\frac{\partial u}{\partial r}+ \frac{\partial ^2 u}{\partial r^2}, \quad r>0, \end{array}\right. } \end{aligned}$$

where the result at $r=0$ is a consequence of l’Hôpital’s rule.

Using a central difference approximation of the Laplacian (35) we produce a semi-descretised approximation to (3) to which we apply a stiff ODE solver (MATLAB’s ode15s function) to obtain numerical solutions $u_\text {Data}(r_j,t_i)$, $v_\text {Data}(r_j,t_i)$, which represent the mobile and bound fluorescent fractions at position $r_j$ and time $t_i$. Then the total fluorescence is

36 $$\begin{aligned} w_\text {Data}(r_j,t_i) = u_\text {Data}(r_j,t_i)+v_\text {Data}(r_j,t_i), \end{aligned}$$

and the fluorescence recovery curve is

37 $$\begin{aligned} F_\text {Data}(t_i) = \frac{1}{\pi r_n^2} \sum _{\lbrace i\in \mathbb {N} \mid r_j\le r_n \rbrace } w_\text {Data}(r_j,t_i) i\delta r. \end{aligned}$$

We will allow for simultaneous fitting of multiple instances of a fluorescence recovery generated using different bleach region radii. Let each of these instances be indexed by a number, $k=1,..., n_\mathrm {exp}$, then let $r_n^k$ be the nominal bleach region radius used in experiment *k*, $w_\text {Data}^k$ the total fluorescence and $F_\text {Data}^k$ (note the superscript *k* does not mean ‘raised to the power of *k*’). We will attempt to fit generated model solutions to synthetic data simulated using known parameter values to ascertain the accuracy of the parameter fitting in various cases.

For each instance *k*, with bleach region radius $r_n^k$, we solve (3) numerically to obtain $u^k(r_j,t_i)$, $v^k(r_j,t_i)$. We define the total fluorescence $w^k(r_j,t_i)$ and the fluorescence recovery curve $F^k(r_j,t_i)$ as in (36) and (37) respectively. We define the objective functions, $\phi$ and $\phi _{\mathrm {Space}}$ as

38 $$\begin{aligned} \phi = \sum _{k=1}^{n_\mathrm {exp}} \left[ \sum _j (F_\text {Data}^k(t_i) - F^k(t_i))^2 \right] , \end{aligned}$$

and

39 $$\begin{aligned} \phi _\text {Space} = \sum _{k=1}^{n_\mathrm {exp}} \left[ \sum _{j} \sum _{i} (w_\text {Data}^k(r_j, t_i) - w^k(r_j,t_i))^2 \right] , \end{aligned}$$

whose minima we attempt to find with the Nelder-Mead downhill simplex algorithm (Nelder and Mead 1965; Olsson and Nelson 1975) (using the fminsearch function of MATLAB). Since we know the values of the parameters used to generate $w_\text {Data}$, we can easily measure the accuracy of the fitting procedure. Let $\theta _l$ be any model parameter ($D_u,D_v,k_\mathrm {on},k_\mathrm {off}$) used to generate $w_\text {Data}$, and $\bar{\theta }_l$ be the fitting procedure output, then the proportional estimation error is

40 $$\begin{aligned} \mu _l^k = \frac{\mid \bar{\theta }_l^k - \theta _l^k \mid }{\theta _l^k}, \end{aligned}$$

where once more the superscript *k* is an index, not a power. The mean error is then simply

41 $$\begin{aligned} \mu _l = \frac{1}{n_\mathrm {run}}\sum _{k=1}^{n_\mathrm {run}} \mu _l^k. \end{aligned}$$

In each case we take $n_\mathrm {run}=1024$. In this way we are able to condense information about the accuracy of parameter estimation in the various dynamic regimes (rapid equilibration, intermediate and so on) into a single variable. However, since $k_\mathrm {on}$ and $k_\mathrm {off}$ may span several orders of magnitude, the mean error $\mu _l$ may be biased by a small number of extreme outlying results. For this reason it may also be of interest to record the number of instances (indexed by *k*), $n_l(\mu )$, which returned values $\bar{\theta }_l^k$ such that $\mu _l^k < \mu$. Then we may define,

42 $$\begin{aligned} f_l(\mu ) = \frac{n_l(\mu )}{n_\text {run}}. \end{aligned}$$

where $\mu$ has a chosen value. For example, if $\mu =0.01$, then $f_l(\mu )$ would be the fraction of instances which returned estimation errors of less than $1\%$.

It is necessary to produce samples of parameter combinations which are used as inputs in generating $w_\text {Data}$, which is done semi-randomly as follows:

1. Generate a uniformly distributed positive random value for $D_u$. We set $D_u\le 50$ $\mu m^2 s^{-1}$ to keep the diffusivity in a biologically realistic range (Kang et al. 2009).
2. Pick a random real number $\tilde{\eta } \in [-3,3]$ from a uniform distribution, and set the dimensionless parameter $\eta =10^{\tilde{\eta }}$. If $\tilde{\eta } \ge 1$, we consider the dynamics to be ‘rapid equilibration’. If $-1<\tilde{\eta } <1$ we consider the dynamics to be ‘intermediate’. Finally, if $\tilde{\eta }\le 1$ we consider the dynamics to be ‘slow equilibration’ (effective diffusion).
3. Set the association rate $k_\mathrm {on}= D_u/(r_n^2 \eta )$.
4. Pick a random real number $\tilde{\kappa } \in [-1,1]$ from a uniform distribution, and set the dimensionless parameter $\kappa =4^{\tilde{\kappa }}$. Cases where $\tilde{\kappa }>1$ or $\tilde{\kappa }<1$ are handled separately.
5. Set the dissociation rate $k_\mathrm {off}= \kappa k_\mathrm {on}$.
6. Pick a random real number $\delta \in [0,10^{-1}]$ from a uniform distribution.
7. Set the slow diffusivity $D_v=\delta D_u$.

Initial guesses are generated in two different ways. First, for the data (recorded in Table 1), each initial guess, $\tilde{\theta }_l^k$, is of the form

43 $$\begin{aligned} \tilde{\theta }_l^k =\theta _l^k (1+ p), \end{aligned}$$

where $p\in [0,0.5]$ is a uniform random variable. This ensures that initial guesses are within $50\%$ of the correct parameter value in each case. This is done mainly to test the predictions in Sect. 3. In the second instance, steps 1-7 were repeated to generate more general random initial guesses (these data are recorded in Table 2).

## Computational results

We begin by considering the rapid equilibration case in which $\eta \gg 1$. On the basis of the analysis in Sect. 3.1.1, we predicted that conventional recovery curve analysis would be generally be sufficient to estimate the slow diffusivity, $D_v$, and the reaction rates, $k_\mathrm {on}, k_\mathrm {off}$. Furthermore, the fast diffusivity, $D_u$, could be estimated given sufficiently high frame rate data. Numerical results (see Table 1) confirm this prediction. We also predicted that the use of spatial data in confocal scanning FRAP would enable the estimation of all of the model parameters, even for a relatively low frame rate, and again our simulated data supports this prediction. The use of spatial data offers a significant improvement over recovery curves alone. On the basis of Table 2, we expect that all four model parameters can be reliably estimated in the $\eta \gg 1$ case by fitting the model to three spatially dynamic fluorescence recoveries with different bleach region radii. We found that this process returned parameter estimates accurate to within $1\%$ of the correct values in at least $92\%$ of instances given initial guesses were also in the $\eta \gg 1$ regime, but otherwise uncontrolled.

**Table 1:** Collated results of parameter fitting tested on synthetic data for precise initial guesses

Regime	Special conditions	$D_u$	$D_v$	$k_\text {on}$	$k_\text {off}$	$D_\text {eff}$
$\eta \gg 1$	–	93%	94%	94%	94%	93%
		(2.2%)	(1.2%)	(0.50%)	(0.43%)	(1.6%)
$\eta \gg 1$	High frame rate	92%	94%	95%	94%	93%
		(2.3%)	(1.6%)	(0.23%)	(0.82)	(1.6%)
$\eta \gg 1$	Spatial	100%	100%	100%	100%	100%
		($1.5\times 10^{-4}$)	($3.2\times 10^{-4}$)	($1.8\times 10^{-4}$)	($1.3\times 10^{-4}$)	($1.1\times 10^{-4}$)
$\eta = O(1)$	High frame rate,	53%	32%	40%	50%	100%
	Spatial	(4.5%)	(37%)	(13%)	(3.2%)	($1.9\times 10^{-2} \%$)
$\eta \ll 1$	–	1.6%	1.6%	0.0%	4.3%	100%
		(24%)	(62%)	(97%)	(14%)	($3.4\times 10^{-3} \%$)
$\eta \ll 1$	High frame rate,	9.8%	1.2%	5.5%	7.8%	100%
	Spatial	(13%)	(51%)	(36%)	(11%)	($5.3\times 10^{-3} \%$)
$\kappa \gg 1$	High frame rate,	98%	0%	8.6%	33%	100%
	Spatial	(0.24%)	(51%)	(43%)	(18%)	($6.7\times 10^{-3} \%$)
$\kappa \ll 1$	High frame rate,	48%	81%	49%	63%	100%
	Spatial	(19%)	(0.80%)	(21%)	(3.4%)	(0.017%)

The first number in each cell is $f_l(0.01)$, defined in (42), that is the fraction of cases which returned results accurate to within $1\%$ of the correct value. The second number in brackets is $\mu _l$, defined in (41), which is the average parameter estimation error. For example, for synthetic FRAP data in the $\eta \gg 1$ regime, in the first row $D_u$ was estimated with an average error of 2.2% and 93% of instanced returned an error of less than 1%. The initial guesses were generated randomly as outlined in Sect. 4, formula (43) of the main text so that each initial guess was within 50% of the correct parameter value. The word ‘spatial’ in the ‘special conditions’ column indicates that the fit was performed using fully spatially dynamic data as in confocal scanning FRAP, otherwise the fit was performed using recovery curves. Likewise, ‘fast frame rate’ indicates that the the time resolution was $\Delta t=10^{-4} s$, otherwise $\Delta t=10^{-2}s$

In the intermediate case ($\eta =O(1)$) we were unable to establish in Sect. 3.1.2 that parameter estimation would be possible unless some parameter values could be determined independently. Numerical results (Table 1) confirm that it is not possible to obtain accurate parameter estimates in most cases, even high frame rate spatial data. Interestingly, however, we consistently found that the effective diffusivity $D_\text {eff}$ was strongly estimable, which suggests that in practice the intermediate fluorescence recovery ($\eta =O(1)$) resembles the effective diffusion recovery ($\eta \ll 1$) quite closely. On the basis of the constraints (32), we expect that improving the resolution of spatially dynamic data would improve parameter estimation by increasing the value of $\eta$. However, since this is not necessarily practical, we also investigated the utility of independently estimating certain parameters, as previous studies have found that fitting multiple fluorescence recoveries with different sized bleach regions (González-Pérez et al. 2011) or independently determining certain model parameters (Sadegh Zadeh et al. 2006) may be beneficial. We therefore investigated the possibility of fitting the reaction rates to data while fixing the diffusivities at some independently determined values. We found (Table 2) that this is method can be used to produce highly accurate estimates of both $k_\mathrm {on}$ and $k_\mathrm {off}$. Supplied with correct values for $D_u$ and $D_v$, and three fluorescence recoveries with different sized bleach regions, we were able to obtain estimates of $k_\mathrm {on}$ and $k_\mathrm {off}$ accurate to within $1\%$ in $100\%$ of instances.

**Table 2:** Collated results of parameter fitting tested on synthetic data for general initial guesses

Regime	Initial guess regime	$D_u$	$D_v$	$k_\text {on}$	$k_\text {off}$	$D_\text {eff}$
$\eta \gg 1$	$\eta \gg 1$	92%	92%	93%	92%	92%
		(14%)	(30%)	(6.0%)	(4.2%)	(10%)
$\eta \gg 1$	$\eta = O(1)$	26%	26%	28%	27%	27%
		(5.1$\times 10^8\%$)	(4.9$\times 10^8\%$)	(7.0$\times 10^8\%$)	(4.0$\times 10^8\%$)	(710%)
$\eta =O(1)$	$\eta =O(1)$	23%	20%	19%	24%	99%
		($2.0\times 10^7\%$)	(120%)	($3.0\times 10^7\%$)	(52%)	(0.11%)
$\eta \ll 1$	$\eta \ll 1$	2.0%	0.39%	0.78%	0.0%	99%
		(66%)	(5.7$\times 10^3\%$)	(890%)	(1.4$\times 10^3\%$)	($8.9\times 10^{-2}\%$)
$\eta \gg 1$	$\eta \gg 1$	–	–	100%	100%	100%
				($7.3\times 10^{-3}\%$)	($5.4\times 10^{-3}\%$)	($1.8\times 10^{-3}\%$)
$\eta =O(1)$	$\eta = O(1)$	–	–	96%	96%	97%
				(5.5%)	(5.0%)	(0.19%)
$\eta =O(1)$	$\eta \gg 1$	–	–	23%	23%	23%
				(77%)	(77%)	(55%)
$\eta \ll 1$	$\eta \ll 1$	–	–	91%	91%	93%
				(540%)	(500%)	(0.29%)

The first number in each cell is $f_l(0.01)$, defined in (42), that is the fraction of cases which returned results accurate to within $1\%$ of the correct value. The second number in brackets is $\mu _l$,defined in (41), which is the average parameter estimation error. For example, for synthetic FRAP data in the $\eta \gg 1$ regime, in the first row $D_u$ was estimated with an average error of 14% and 92% of instanced returned an error of less than 1%. The initial guesses were generated randomly as outlined in Sect. 4, so that only the regime (e.g. $\eta \gg 1$) was known

In Sect. 3.1.3 we predicted that in the $\eta \ll 1$ case it will not be possible to identify individual parameter values, only to show that they lie within a manifold defined by

44 $$\begin{aligned} \frac{k_\mathrm {on}D_v + k_\mathrm {off}D_u}{k_\mathrm {off}+ k_\mathrm {on}} = D_\text {eff}, \end{aligned}$$

for constant $D_\text {eff}$. We found that it is possible to estimate accurately the effective diffusivity $D_\text {eff}$, but not of any of the parameters individually (see Table 1). In accordance with our predictions, we did not find that increasing frame rate or the use of spatial data, unless of extremely high resolution, could improve this (Table 2). As in the $\eta =O(1)$ case, it will be necessary to estimate the diffusivities $D_u$ and $D_v$ separately; however, unlike the $\eta =O(1)$ case, this does not enable us to estimate the reaction rates, $k_\mathrm {on}$ and $k_\mathrm {off}$, only the ratio $\kappa = k_\mathrm {off}/k_\mathrm {on}$. The estimation accuracy of $\kappa$ is closely depends upon the estimation accuracy of $D_u$ and $D_v$, with the relationship between them being

45 $$\begin{aligned} \kappa = \frac{D_u-D_\text {eff}}{D_\text {eff}-D_v}. \end{aligned}$$

Finally, we considered the case of asymmetric reaction rates, $\kappa \gg 1$ and $\kappa \ll 1$. As predicted in Sects. 3.1.4 and 3.1.5 only $D_u$ and $D_v$ respectively are measurable in this case. Increasing frame rate, fitting multiple fluorescence recoveries, and using spatial data regardless of resolution are not beneficial (Table 1).

Our summarised results are as follows:

- When $\eta \gg 1$, all model parameters can be estimated. This may be possible with conventional analysis of recovery curves, but it is more reliable to fit a spatially dynamic model to confocal FRAP data.
- When $\eta = O(1)$, $k_\mathrm {on}$ and $k_\mathrm {off}$ can be estimated. To do this, it necessary to conduct separate experiments in order to measure $D_u$ and $D_v$ accurately.
- When $\eta \ll 1$, the ratio $k_\mathrm {off}/k_\mathrm {on}$ can be estimated. As in the previous case, it necessary to conduct separate experiments in order to measure $D_u$ and $D_v$ accurately.
- When $k_\mathrm {off}/k_\mathrm {on}\gg 1$ or $k_\mathrm {off}/k_\mathrm {on}\ll 1$, it is only possible to measure $D_u$ or $D_v$ respectively. There is no experiment which could reliably determine $k_\mathrm {on}$, $k_\mathrm {off}$ or the ratio of the two.

## Regime identification

We have so far determined that the reliability and accuracy of parameter estimation are determined by the parameter regime of the data. However, one does not automatically know the regime of experimental data. The objective of this section is therefore to determine the precise boundary between the regimes and propose a method to determine the regime of arbitrary FRAP data. To this end, we ran numerical experiments in which we attempted fitting on synthetic data with procedurally generated parameter inputs, as described in Sect. 4, but precisely controlling the values of the dimensionless quantities, $\eta$ and $\kappa$. We consider $\eta$ in Sect. 6.1 and $\kappa$ in Sect. 6.2.

### The effect of varying $\eta$

In order to locate the boundary between the regimes we ran a sample of parameter fitting experiments with $\eta$ values in a set of intervals, $\eta \in [10^{\tilde{\eta }},10^{\tilde{\eta }+0.1}]$ with $\tilde{\eta }\in \lbrace ..., -0.2, -0.1, 0,0.1,0.2, ... \rbrace$, and recorded the fraction of output parameter estimates with an error of less than $1\%$ relative to the correct corresponding parameter input value ($f_l(0.01)$ as defined in (42)).

**Fig. 6:**
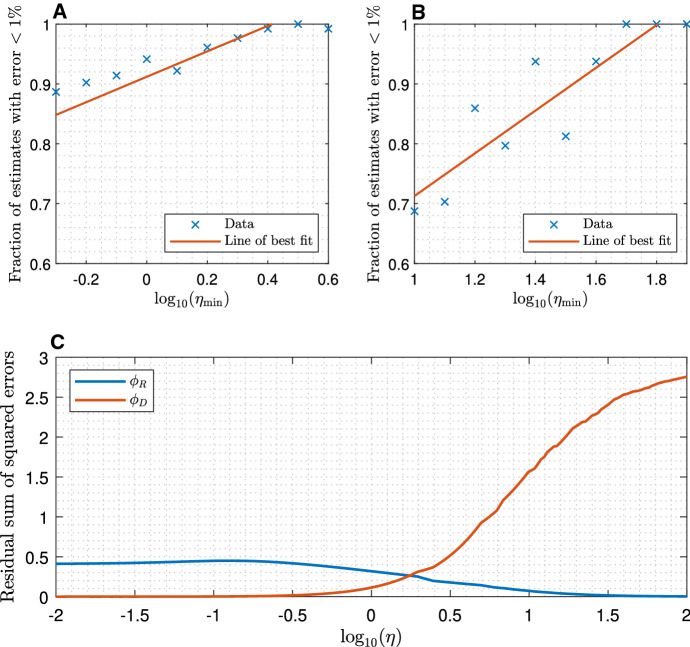
**a** Fraction of parameter estimates within $1\%$ of the correct value ($f_l(0.01)$ as defined in (42)) when attempting to fit just $k_\mathrm {on}$ and $k_\mathrm {off}$. Each data point is calculated from $n_\mathrm {run}=128$ instances of fitting with $\eta \in [\eta _\mathrm {min},\eta _\mathrm {max}]$ where $\eta _\mathrm {min}$ is indicated and $\log _{10}(\eta _\mathrm {max}) = \log _{10}(\eta _\mathrm {min})+0.1$. **b** identical to **a**, except attempting to fit $D_u$, $D_v$, $k_\mathrm {on}$ and $k_\mathrm {off}$. **c** Residual sum of squared errors between a simulated fluorescence recovery curve and recovery curves computed by the rapid equilibration formula (16) ($\phi _R$) and the slow equilibration (effective diffusion) formula (21) ($\phi _D$). Parameters were $D_u=30$ $\mu m^2 s^{-1}$, $D_v=0.01$ $\mu m^2 s^{-1}$, $r_n = 0.5$ $\mu m$, $k_\mathrm {on}= k_\mathrm {off}= D_u / (r_n^2\eta )$ for variable $\eta$. The rapid equilibration error, $\phi _R$, decreases as $\eta$ increases, while the effective diffusion error, $\phi _D$, increases

Results (Fig. 6) indicated that, as expected, the reliability of the fit generally increased with the value of $\eta$. When $D_u$ and $D_v$ were known, fits of the reaction rates $k_\mathrm {on}$ and $k_\mathrm {off}$ were consistently accurate for $\eta > 10^{0.4}\approx 2.51$ (Fig. 6a). Fitting all four parameters reliably, however, required $\eta >10^{1.7}\approx 50.1$ (Fig. 6b).

We would expect that, in the regime where accurate estimation of all model parameters is possible, the rapid equilibration formula (16) ought to well-approximate the recovery curve. Accordingly, it is clear in Fig. 6c that the error between formula (16) and simulated data, $\phi _R$, decreases as $\eta$ increases, and is negligible for $\eta > 10^{1.7}$. Similarly, we would expect the slow equilibration (effective diffusion) formula (21) to be a good approximation where estimation of $k_\mathrm {on}$ and $k_\mathrm {off}$ is not possible. Although the error, $\phi _D$, decreases as $\eta$ decreases, as we would expect, it does not appear that the effective diffusion formula is a good approximation when $\eta = 10^{0.4}$. This suggests that for $\eta \approx 1$, neither the effective diffusivity $D_\mathrm {eff}$, nor $k_\mathrm {on}$ and $k_\mathrm {off}$ individually, are estimable with total accuracy.

We can place data into one of three regimes: rapid equilibration ($\eta > 10^{1.7}$), intermediate ($10^{0.4}\le \eta \le 10^{1.7}$), and slow equilibration $\eta <10^{0.4}$. If the regime can be determined, then the required course of action is obvious: in the rapid equilibration regime full parameter fitting is possible, in the intermediate regime the reaction rates can be estimated after separate experiments to determine the diffusivities have been conducted, while in the slow equilibration regime at most the ratio of the reaction rates can be estimated.

We propose that the regime can be identified by attempting separate fits which are restricted to particular regimes. The best fit corresponds to the correct regime of the data. We measured goodness-of-fit with the Akaike information criterion (Akaike 1998),

46 $$\begin{aligned} \mathrm {AIC} = 2N_\mathrm {param} + N_\mathrm {data}\log (\phi ), \end{aligned}$$

where $N_\mathrm {param}$ is the number of model parameters, $N_\mathrm {data}$ is the number of data points and $\phi$ is the objective function/residual sum of squared errors. The model with the smallest AIC is in general the best fit with the least degree of over-fitting.

We tested procedurally generated data by fitting in the three major model regimes, as well as by fitting with a pure diffusion model. The restricted parameter estimation was implemented using MATLAB’s constrained optimisation algorithm, fmincon. We define $\mathrm {AIC}_\mathrm {RE}$ as the AIC resulting from a model fit which is limited to the rapid equilibration regime, while $\mathrm {AIC}_\mathrm {I}$ and $\mathrm {AIC}_\mathrm {SE}$ are likewise for the intermediate regime and the slow equilibration regime respectively. $\mathrm {AIC}_\mathrm {D}$ is the AIC of the pure diffusion model fit. Note that $N_\mathrm {param} = 4$ for $\mathrm {AIC}_\mathrm {RE}$, $\mathrm {AIC}_\mathrm {I}$ and $\mathrm {AIC}_\mathrm {SE}$, while $N_\mathrm {param}=1$ for $\mathrm {AIC}_\mathrm {D}$. For this reason, the pure diffusion model will yields a lower AIC than the full reaction–diffusion model in cases where the residual sum of squared errors, $\phi$, are equal.

**Table 3:** Regime identification with constrained parameter fitting tested on a sample of 128 synthetic experiments for each regime

X (regime)	$\mathrm {AIC}_\mathrm {X} < \mathrm {AIC}_\mathrm {RE}$	$\mathrm {AIC}_\mathrm {X} < \mathrm {AIC}_\mathrm {I}$	$\mathrm {AIC}_\mathrm {X} < \mathrm {AIC}_\mathrm {SE}$	$\mathrm {AIC}_\mathrm {X} < \mathrm {AIC}_\mathrm {D}$
Large $\eta$ (RE)	–	$100\%$	$100\%$	$100\%$
Intermediate (I)	$100\%$	–	$100\%$	$100\%$
Small $\eta$ (SE)	$100\%$	$100\%$	–	$84.4\%$

The leftmost row indicates the regime of the data: rapid equilibration (RE), intermediate (I) and slow equilibration (SE). Each cell displays the percentage of cases in which the AIC in a regime indicated by the column was greater than the AIC in the correct regime indicated by the row

Results in Table 3 indicate that, for both intermediate and rapid equilibration, constrained fitting in the correct regime produced the best fit in all cases, which strongly supports our contention that this method can be used for regime identification. In a minority of cases, the pure diffusion model provided a better fit than the full model in the slow equilibration regime, hence a slow equilibration recovery cannot reliably be distinguished from a purely diffusive recovery. This is to be expected, as the fluorescence recovery in slow equilibration regime tends to resemble pure diffusion with effective diffusivity, $D_\mathrm {eff}$.

### The effect of varying $\kappa$

**Fig. 7:**
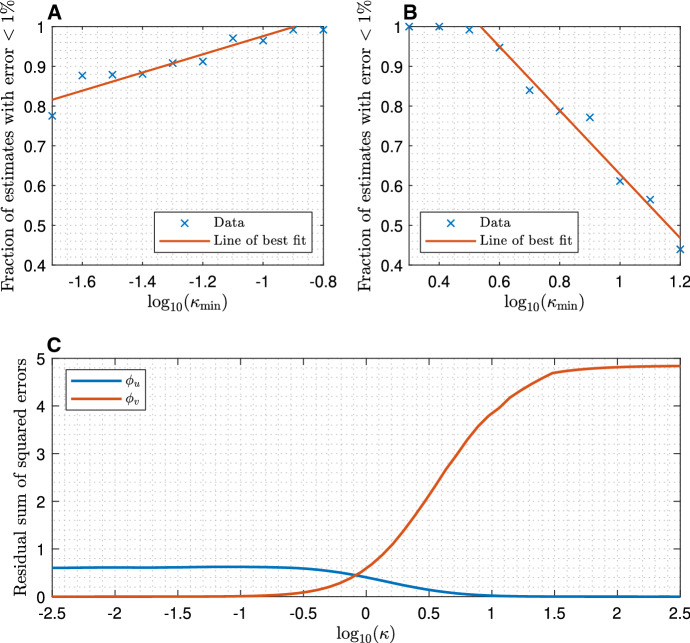
**a** Fraction of parameter estimates within $$1\%$$ of the correct value ($$f_l(0.01)$$ as defined in (42)) when attempting to fit $$D_u$$, $$D_v$$, $$k_\mathrm {on}$$ and $$k_\mathrm {off}$$. Each data point is calculated from $$n_\mathrm {run}=128$$ instances of fitting with $$\kappa \in [\kappa _\mathrm {min},\kappa _\mathrm {max}]$$ where $$\kappa _\mathrm {min}$$ is indicated and $$\log _{10}(\kappa _\mathrm {max}) = \log _{10}(\kappa _\mathrm {min})+0.1$$. **b** Similar to **a**, except over a different range of values of $$\kappa _\mathrm {min}$$. **c** Residual sum of squared errors between a simulated fluorescence recovery curve and recovery curves computed by the pure diffusion formula (23) with diffusivities $$D_u$$ ($$\phi _u$$) and $$D_v$$ ($$\phi _v$$). Parameters were $$D_u=8$$ $$\mu m^2 s^{-1}$$, $$D_v=1$$ $$\mu m^2 s^{-1}$$, $$r_n = 0.5$$ $$\mu m$$, $$k_\mathrm {on}= 1$$ $$s^{-1} $$ and $$k_\mathrm {off}= \kappa k_\mathrm {on}$$ for variable $$\kappa $$. The goodness-of-fit of pure diffusion with diffusivity $$D_u$$ improves as $$\kappa $$ increases, while the fit with diffusivity $$D_v$$ improves as $$\kappa $$ decreases

As with $\eta$, we began investigating the effect of varying $\kappa$ on parameter estimation by locating the boundary between the regimes. Computational results (Fig. 7a, b) indicate that parameter estimation deteriorates the further $\kappa$ deviates from 1 in either direction. We found that $10^{-0.9}< \kappa < 10^{0.53}$ ensured reliably accurate estimation of all four model parameters.

As $\kappa \rightarrow \infty$, the system asymptotically approaches a pure diffusion recovery with diffusivity $D_u$, and likewise for $D_v$ as $\kappa \rightarrow 0$. Yet the pure diffusion model with the appropriate diffusivity is a better approximation for $\kappa = 10^{-0.9}$ than for $\kappa = 10^{0.53}$ (Fig. 7c). We believe that this asymmetry can be explained as follows. Since $D_u>D_v$, the diffusive recovery with diffusivity $D_u$ is faster, hence there are comparatively fewer data points available with the fluorescence recovery in progress, ultimately leading to less accurate parameter estimates.

Next, we tested whether constrained fitting can identify the magnitude of $\kappa$, similar to $\eta$ in Sect. 6.1. Again, we computed the Akaike information criterion of various fits limited to different regimes: $\mathrm {AIC}_\mathrm {U}$ for $\kappa >10^{0.53}$, $\mathrm {AIC}_\mathrm {V}$ for $\kappa <10^{-0.9}$, and $\mathrm {AIC}_\mathrm {D}$ for the pure diffusion model. For fits where $\kappa$ was of intermediate magnitude ($10^{-0.9}<\kappa <10^{0.53}$), we also imposed $\eta >10^{1.7}$ (*i.e.* the rapid equilibration regime considered in Sect. 6.1). We made this imposition because rapid equilibration is the sole regime in which full parameter estimation is possible, so identifying it is the most important problem.

**Table 4:** Regime identification with constrained parameter fitting tested on a sample of 128 synthetic experiments for each regime

X (regime)	$\mathrm {AIC}_\mathrm {X} < \mathrm {AIC}_\mathrm {U}$	$\mathrm {AIC}_\mathrm {X} < \mathrm {AIC}_\mathrm {RE}$	$\mathrm {AIC}_\mathrm {X} < \mathrm {AIC}_\mathrm {V}$	$\mathrm {AIC}_\mathrm {X} < \mathrm {AIC}_\mathrm {D}$
Large $\kappa$ (U)	–	$100\%$	$82.0\%$	$95.3\%$
Intermediate $\kappa$ (RE)	$100\%$	–	$100\%$	$100\%$
Small $\kappa$ (V)	$89.1\%$	$88.3\%^*$	–	$100\%$

The leftmost row indicates the regime of the data: $\kappa >10^{0.53}$ (U), rapid equilibration with intermediate $\kappa$ (RE), and $\kappa <10^{-0.9}$ (V). Each cell displays the percentage of cases in which the AIC in a regime indicated by the column was greater than the AIC in the correct regime indicated by the row

Results (Table 4) clearly indicate that the $\kappa \gg 1$ and $\kappa \ll 1$ regimes cannot always be distinguished from one another, nor can they always be distinguished from pure diffusion; however this is unavoidable as both regimes are approximately diffusive.

For rapid equilibration data, the fit constrained to the rapid equilibration regime gave the best fit in all cases, which encouragingly suggests that this regime can be identified. On the other hand, for $\kappa \ll 1$ data, the fit constrained to the rapid equilibration regimes gave a better fit in $11.7\%$ of cases. Judging by goodness-of-fit alone, we would erroneously conclude that these data were rapid equilibration, leading to potentially wildly inaccurate parameter estimates. However, in all of these instances we had $\bar{\kappa } \le 10^{-0.9} + 10^{-3}$ where $\bar{\kappa }$ is the estimated value of $\kappa$. The algorithm clearly converged towards a point which was as close as possible to the $\kappa \ll 1$ regime (the correct regime). We therefore imposed the additional rule that a regime is not considered viable if the constrained fit in that regime yields parameter estimates at the boundary between regimes. With the addition of this rule, in all of our numerical tests we were able to identify the rapid equilibration regime without any false positives or false negatives.

It is worthwhile noting that, even though the fluorescence recovery approximates pure diffusion as $\kappa \rightarrow \infty$ or $\kappa \rightarrow 0$, the $\kappa \gg 1$ and $\kappa \ll 1$ regimes could not be reliably identified with model selection alone. For $\kappa \gg 1$ and $\kappa \ll 1$, we found that $\mathrm {AIC}_\mathrm {RE}<\mathrm {AIC}_\mathrm {D}$ in $63.3\%$ and $74.2\%$ of cases respectively. In other words, the reaction–diffusion model produced a better fit than the pure diffusion model in the majority of cases. It is clear, then, that constrained fitting of the reaction–diffusion model is essential for the purposes of regime identification.

### The diffusive regimes, $\eta \ll 1$, $\kappa \gg 1$ and $\kappa \ll 1$

Although the $\eta \gg 1$ and $\eta =O(1)$ regimes can be identified, the $\kappa \ll 1$, $\kappa \gg 1$ and $\eta \ll 1$ regimes cannot be differentiated from one another as they all somewhat resemble diffusive recoveries. However, this is no problem, as these regimes can easily be identified by other means. Suppose that *D* is optimum diffusivity obtained from fitting the pure diffusion model to data. If $D\approx D_u$ then $\kappa \gg 1$ and $v_\mathrm {eq}\approx 0$, while if $D \approx D_v$ then $\kappa \ll 1$ and $v_\mathrm {eq}\approx 1$. If it is clear that $D_v<D<D_u$, then $D=D_\mathrm {eff}$ and $\kappa$ can be calculated using formula (45). In summary, it is always possible, in principle, to determine the parameter regime, and by extension, which parameters are estimable and under what circumstances, of given FRAP data.

## Discussion

The application of mathematical modelling to FRAP can improve the understand of biological systems by enabling researchers to extract quantitative binding information from fluorescence microscopy data. In this article we investigated the feasibility of obtaining quantitative information from fluorescence microscopy data. On the basis of approximations derived using formal asymptotic methods, we theoretically predicted the conditions under which a FRAP inverse modelling problem (the problem of determining parameter values from data) is tractable in terms of biological and experimental parameters. We found that, in all cases, the inverse modelling problem is tractable only if

47 $$\begin{aligned} \frac{k_\mathrm {off}}{k_\mathrm {on}} =O(1), \quad D_v \ll D_u. \end{aligned}$$

For conventional FRAP recovery curve analysis we predicted that the following sufficient conditions ensure tractability:

48 $$\begin{aligned} \frac{D_u}{k_\mathrm {on}r_n^2}\ll 1, \Delta t \ll \frac{r_n^2}{D_u}. \end{aligned}$$

where $\Delta t$ is the temporal resolution of the data and $r_n$ is the radius of the bleach region. Since many modern FRAP experiments are carried out using confocal scanning laser microscopy, we also considered the use of spatial information in FRAP fitting, and derived the following sufficient conditions for tractability

49 $$\begin{aligned} \frac{D_u}{k_\mathrm {on}\Delta r^2}\gg 1, \frac{D_u}{k_\mathrm {on}L^2} \lesssim 1, \end{aligned}$$

where $\Delta r$ is the length scale of a single pixel and *L* is the length scale of the whole of the imaged region.

Whenever the rates of molecular association and dissociation are of comparable order, all FRAP model parameters may be inferred from either conventional FRAP or confocal scanning FRAP data of sufficient temporal and/or spatial resolution. We expect that this will the case in many circumstances, but not universally. We found (Sect. 5) that when the tractability conditions are not met, it is still possible to estimate the reaction rates $k_\mathrm {on}$ and $k_\mathrm {off}$, or at the very least the ratio $k_\mathrm {off}/k_\mathrm {on}$, by estimating the diffusivities $D_u$ and $D_v$ independently. We also proposed simple tests to determine when full parameter fitting is possible and when separate experiments will be required.

Despite the large number of quantitative FRAP studies which have been published, in practice researchers have often preferred to fit recovery curves with a simple exponential formula, even in cases where pure diffusion is likely the best model of the system under investigation (Taylor et al. 2019). Even in rapid equilibration reaction–diffusion systems with $D_v=0$, where the exponential formula is appropriate, it is nevertheless an under-utilisation of data, as it yields only an estimate of the dissociation rate, $k_\mathrm {off}$, where estimates of the association rate, $k_\mathrm {on}$, and diffusivity $D_u$ are possible. Yet, the exponential formula is not really applicable to a diffusion-based recovery, and it must be noted that inappropriate model choice may lead to inaccurate parameter estimates and incorrect conclusions (Sprague et al. 2004; Mueller et al. 2010; Mazza et al. 2012). Therefore, it is our belief that a thorough approach to FRAP parameter estimation, incorporating model selection and regime identification, would be beneficial. It is our intention to develop this approach in future work, utilising the theoretical results which we have established here.

## Derivations

In this appendix we present the derivations of the formulae in Sect. 3. We begin with the non-dimensionalised FRAP equation,

50 $$\begin{aligned} {\left\{ \begin{array}{ll} \frac{\partial u}{\partial t'} = -u + \kappa v + \eta \nabla '^2u, \\ \frac{\partial v}{\partial t'} = u - \kappa v + \delta \eta \nabla '^2v, \end{array}\right. } \end{aligned}$$

which is equation (5) in the main text. Equation (50) is subject to the the far-field conditions

51 $$\begin{aligned} \lim _{r\rightarrow \infty }u(r',t')=u_\mathrm {eq}= \frac{\kappa }{1+ \kappa }, \lim _{r \rightarrow \infty }v(r',t')=v_\mathrm {eq}= \frac{1}{1+\kappa }, \end{aligned}$$

and the post-bleach initial conditions,

52 $$\begin{aligned} u(r,0) = u_\mathrm {eq}H(r'-1), \quad v(r,0) = v_\mathrm {eq}H(r'-1), \end{aligned}$$

where *H* is the Heaviside step function. The dimensionless variables are

53 $$\begin{aligned} r'= \frac{r}{r_n}, \quad t'=k_\mathrm {on}t, \end{aligned}$$

and the dimensionless parameters are

54 $$\begin{aligned} \eta = \frac{D_u}{r_n^2 k_\mathrm {on}}, \quad \kappa = \frac{k_\mathrm {off}}{k_\mathrm {on}}, \quad \delta = \frac{D_v}{D_u}. \end{aligned}$$

By assumption the Laplacian is radially symmetric

55 $$\begin{aligned} \nabla '^2u = \frac{1}{r'}\frac{\partial }{\partial r'} \left( r' \frac{\partial u}{\partial r'} \right) = r_n^2 \nabla ^2u = r_n^2 \frac{1}{r}\frac{\partial }{\partial r} \left( r\frac{\partial u}{\partial r} \right) , \end{aligned}$$

and likewise for *v*. We will use the following asymptotic expansion in the subsequent sections,

56 $$\begin{aligned} {\left\{ \begin{array}{ll} u(r',t')=u_0 (r',t') + \varepsilon u_1(r',t') + \varepsilon ^2 u_2 (r',t') + ... ,\\ v(r',t')=v_0 (r',t') + \varepsilon v_1(r',t') + \varepsilon ^2 v_2 (r',t') + ... . \end{array}\right. } \end{aligned}$$

### Rapid equilibration ($\eta \gg 1$)

In the first instance, we will derive the rapid equlibration recovery curve formula, (16) in the main text. We set $\varepsilon = 1/\eta$. Substituting expansion (56) into system (50) yields

57 $$\begin{aligned} {\left\{ \begin{array}{ll} \varepsilon \frac{\partial u_0}{\partial t'} = \varepsilon (\kappa v_0 - u_0) + \nabla '^2u_0 + \varepsilon \nabla '^2u_1 + O(\varepsilon ^2),\\ \frac{\partial v_0}{\partial t'} + \varepsilon \frac{\partial v_1}{\partial t'} = (u_0-\kappa v_0) + \varepsilon (u_1-\kappa v_1) +\frac{\delta }{\varepsilon }\nabla '^2v_0 + \delta \nabla '^2v_1 + \delta \varepsilon \nabla '^2v_2 +O(\varepsilon ^2). \end{array}\right. } \end{aligned}$$

We will necessarily be left with $\nabla '^2u_0 = \nabla '^2v_0 = 0$ as we let $\varepsilon \rightarrow 0$ unless we also take $\delta \rightarrow 0$ (that is, the entire system instantaneously returns to equilibrium in the limiting case where both species are infinitely diffusive). Neglecting this uninteresting case, taking the limit $\varepsilon ,\delta \rightarrow 0$ such that $\delta /\varepsilon = O(1)$, we are left with a single equation in $v_0$,

58 $$\begin{aligned} \frac{\partial v_0}{\partial t'} = u_\mathrm {eq}-\kappa v_0 + \frac{\delta }{\varepsilon }\nabla '^2v_0, \end{aligned}$$

where we have already used the fact that $u_0=u_\mathrm {eq}$, since this is the unique solution of $\nabla '^2u_0=0$ given the boundary conditions (51). Noting that $k_\mathrm {on}u_\mathrm {eq}=k_\mathrm {off}v_\mathrm {eq}$ and making the substitution $v_0(r,t)=v_\mathrm {eq}- \tilde{v}_0(r,t)$, we arrive at

59 $$\begin{aligned} \frac{\partial \tilde{v}_0}{\partial t} = -k_\mathrm {off}\tilde{v}_0 + D_v \nabla ^2\tilde{v}_0, \end{aligned}$$

where we should note that (59) has been restored to dimensional form. The solution to (59) subect to Dirac delta intitial conditions is

60 $$\begin{aligned} \tilde{v}(r,t)= \frac{1}{4\pi D_v t} \exp \left( -k_\mathrm {off}t\right) \exp \left( - \frac{r^2}{4 D_v t}\right) , \end{aligned}$$

hence the solution to the general initial value problem of (59) is, in Cartesian coordinates,

61 $$\begin{aligned} \tilde{v}(x,y,t)=\iint _{\mathbb {R}^2} \tilde{v}(x',y',0)\frac{1}{4\pi D_v t} \exp \left( -k_\mathrm {off}t - \frac{(x'-x)^2 + (y'-y)^2}{4 D_v t}\right) \text {d}x \text {d}y, \end{aligned}$$

which implies that

62 $$\begin{aligned} \tilde{v}(x,y,t)e^{ k_\mathrm {off}t }=\frac{v_\mathrm {eq}}{4\pi D_v t} \iint _{\mathbb {R}^2} \left( 1-H(x'^2+y'^2-r_n^2) \right) \exp \left( - \frac{(x'-x)^2 + (y'-y)^2}{4 D_v t}\right) \text {d}x \text {d}y. \end{aligned}$$

At this stage, the derivation becomes virtually identical to that of Soumpasis (1983). The precise form of $v_0$ can only be expressed in integral form, yet if we define the contribution of the bound fraction to the fluorescence recovery as

63 $$\begin{aligned} F_v(t) = \frac{1}{\pi r_n^2}\int _0^{2\pi }\int _0^{r_n} v_0 (r,t)\text {d}r\text {d}\theta , \end{aligned}$$

then we conclude that

64 $$\begin{aligned} F_v(t)=v_\mathrm {eq}- v_\mathrm {eq}e^{-k_\mathrm {off}t} \left( 1 - \mathscr {F}_S \left( \frac{r_n^2}{2D_v t} \right) \right) . \end{aligned}$$

The above approximation breaks down over the short time scale, $t'=O(\varepsilon )$, so it is helpful to consider the rescaling $t'=\varepsilon \tau$ which, in the limit $\varepsilon \rightarrow 0$ reduces (57) to

65 $$\begin{aligned} \frac{\partial u_0}{\partial \tau }=\nabla '^2u_0, \quad \frac{\partial v_0}{\partial \tau } = 0, \end{aligned}$$

which indicates that the dynamics of *u* are governed purely by diffusion, implying that, if we define $F_u$ analogously to $F_v$ such that

66 $$\begin{aligned} F_u(t) = \frac{1}{\pi r_n^2}\int _0^{2\pi }\int _0^{r_n} u_0 (r,t)\text {d}r\text {d}\theta , \end{aligned}$$

we conclude that

67 $$\begin{aligned} F_u(t) = u_\mathrm {eq}\mathscr {F}_S\left( \frac{r_n^2}{2D_u t} \right) . \end{aligned}$$

Finally, noting that the total fluorescence recovery curve is given by $F(t)=F_u(t)+F_v(t)$ and that $u_\mathrm {eq}=k_\mathrm {off}/(k_\mathrm {on}+k_\mathrm {off})$, $v_\mathrm {eq}=k_\mathrm {on}/(k_\mathrm {on}+k_\mathrm {off})$, we arrive at

68 $$\begin{aligned} F(t)=\frac{k_\mathrm {off}}{k_\mathrm {on}+k_\mathrm {off}}\mathscr {F}_S\left( \frac{r_n^2}{2 D_u t}\right) + \frac{k_\mathrm {on}}{k_\mathrm {on}+k_\mathrm {off}} \left( 1 - e^{-k_\mathrm {off}t}\left[ 1 - \mathscr {F}_S\left( \frac{r_n^2}{2 D_v t}\right) \right] \right) . \end{aligned}$$

### Intermediate equilibration ($\eta =O(1)$)

We now derive FRAP formula (20) from the main text by taking the asymptotic expansion (57) to first order. We can eliminate the ‘zeroth’ order, which we have already balanced, where we cancel $\varepsilon$ throughout. Then, once again considering the limit $\delta , \varepsilon \rightarrow 0$, we are left with

69 $$\begin{aligned} {\left\{ \begin{array}{ll} \nabla '^2u_1 = u_0 - \kappa v_0 ,\\ \frac{\partial v_1}{\partial t'} = + (u_1-\kappa v_1) + \frac{\delta }{\epsilon } \nabla '^2v_1. \end{array}\right. }. \end{aligned}$$

Since, $u_0$ and $v_0$ entirely satisfy the far field conditions (51), for consistency we impose

70 $$\begin{aligned} \lim _{r\rightarrow \infty }u_1(r',t')=0, \lim _{r \rightarrow \infty }v_1(r',t')= 0. \end{aligned}$$

When $r'\ge 1$, we have $v_0(r',t')=v_\mathrm {eq}$, hence $\nabla '^2u_1 = u_\mathrm {eq}- \kappa v_\mathrm {eq}= 0$, which taken together with (70) implies that $u_1(r','t)=0$ for $r'\ge 1$. Imposing the continuity condition $u_1(1^{-},t')=0$, we then solve for $u_1$ on $r'\le 1$

71 $$\begin{aligned} \frac{1}{r'}\frac{\partial }{\partial r'}\left( r'\frac{\partial u_1}{\partial r'} \right) = u_\mathrm {eq}- \kappa v_\mathrm {eq}(1-e^{-\kappa t'}) = u_\mathrm {eq}e^{-\kappa t'}, \end{aligned}$$

which implies that

72 $$\begin{aligned} u_1 (r',t') = \frac{1}{4}u_\mathrm {eq}e^{-\kappa t'} ({r'}^2 - 1), \end{aligned}$$

where we have used the symmetry condition $\left. \frac{\partial u_1}{\partial r'} \right| _{r=0} = 0$. Further analytic progress is possible in the limiting case $\delta /\varepsilon \rightarrow 0$, by multiplying through (69) with the integrating factor $e^{\kappa t'}$, yielding

73 $$\begin{aligned} e^{\kappa t'} \frac{\partial v_1}{\partial t'} + \kappa e^{\kappa t'} v_1 = \frac{\partial }{\partial t'}(v_1 e^{\kappa t'}) = \frac{1}{4}u_\mathrm {eq}({r'}^2 - 1). \end{aligned}$$

Taking $v_1(r',0)=0$ as the initial condition, we are left with

74 $$\begin{aligned} v_1 (r',t') = \frac{1}{4}u_\mathrm {eq}({r'}^2 - 1) t'e^{\kappa t'}. \end{aligned}$$

We may precisely express the asymptotic expansion as follows

75 $$\begin{aligned} {\left\{ \begin{array}{ll} u(r,t)= u_\mathrm {eq}+ \varepsilon \frac{1}{4}u_\mathrm {eq}e^{-\kappa t'} ({r'}^2 - 1) + O(\varepsilon ^2),\\ v(r,t) = v_\mathrm {eq}(1-e^{\kappa t'}) + \varepsilon \frac{1}{4}u_\mathrm {eq}t'e^{-\kappa t'} ({r'}^2 -1) + O(\varepsilon ^2). \end{array}\right. } \end{aligned}$$

In dimensional form and truncated to first order, we have

76 $$\begin{aligned} {\left\{ \begin{array}{ll} u(r,t) = u_\mathrm {eq}\left( 1 + \frac{k_\mathrm {on}}{4D_u} e^{-k_\mathrm {off}t}(r^2 - r_n^2) \right) ,\\ v(r,t) = v_\mathrm {eq}\left( 1 - e^{-k_\mathrm {off}t} + \frac{k_\mathrm {on}}{4D_u} k_\mathrm {off}t e^{-k_\mathrm {off}t} (r^2 - r_n^2) \right) . \end{array}\right. } \end{aligned}$$

We are now able to evaluate the fluorescence recovery function,

77 $$\begin{aligned} F(t)=\frac{1}{\pi r_n^2}\int _0^{2\pi }\int _0^{r_n} (u(r,t)+v(r,t))\text {d}r \text {d}t, \end{aligned}$$

to obtain

78 $$\begin{aligned} F(t) = 1-v_\mathrm {eq}e^{-k_\mathrm {off}t} -\frac{k_\mathrm {on}r_n}{3 D_u} e^{-k_\mathrm {off}t}(1+k_\mathrm {off}t), \end{aligned}$$

as required.

### Slow equilibration ($\eta \ll 1$)

Finally, we will derive the FRAP formula (21) from the main text. Let $\varepsilon = \eta$. We begin by truncating the asymptotic expansions, (56), of *u* and *v* to zeroth order prior to substitution into (50), yielding

79 $$\begin{aligned} {\left\{ \begin{array}{ll} \frac{\partial u_0}{\partial t'} = \kappa v_0 - u_0 + \epsilon \nabla '^2u_0,\\ \frac{\partial v_0}{\partial t'} = u_0 - \kappa v_0 + \delta \epsilon \nabla '^2v_0, \end{array}\right. }\end{aligned}$$

In the limit $\varepsilon \rightarrow 0$, there is no net spatial redistribution and the the total concentration, $w_0=u_0+v_0$ is time-invariant, yet it is clear that the system will converge towards a local chemical equilibrium at each point, given by $u_0 = \kappa w_0/(1+\kappa ), v_0=w_0/(1+\kappa )$. Notwithstanding, this approximation fails over sufficiently long time scales ($t'=O(1/\varepsilon )$), which we may investigate by rescaling time such that we have $t'=\tau /\varepsilon$ for $\tau = O(1)$, yielding

80 $$\begin{aligned} {\left\{ \begin{array}{ll} \varepsilon \frac{\partial u_0}{\partial \tau }(r',\tau /\varepsilon ) = \kappa v_0(r',\tau /\varepsilon ) - u_0(r',\tau /\varepsilon ) + \varepsilon \nabla '^2u_0(r',\tau /\varepsilon ),\\ \varepsilon \frac{\partial v_0}{\partial \tau }(r',\tau /\varepsilon ) = u_0(r',\tau /\varepsilon ) - \kappa v_0(r',\tau /\varepsilon ) + \delta \varepsilon \nabla '^2v_0 (r',\tau /\varepsilon ). \end{array}\right. } \end{aligned}$$

Adding the two equations in system (80) neutralises the zeroth order terms

81 $$\begin{aligned} \frac{\partial u_0}{\partial \tau } + \frac{\partial v_0}{\partial \tau } = \nabla '^2u_0(r',\tau /\epsilon )+ \delta \nabla '^2v_0 (r',\tau /\epsilon ), \end{aligned}$$

where we have cancelled $\varepsilon$ throughout. Taking $\varepsilon \rightarrow \infty$

82 $$\begin{aligned} \frac{\partial w_0}{\partial \tau } = \left( \frac{\kappa }{1+\kappa }+\frac{\delta }{1+\kappa } \right) \nabla '^2w_0. \end{aligned}$$

At this point it is convenient to re-dimensionalise, finally yielding

83 $$\begin{aligned} \frac{\partial w_0}{\partial t} = \frac{k_\mathrm {off}D_u + k_\mathrm {on}D_v}{k_\mathrm {on}+k_\mathrm {off}}\nabla ^2w_0 = D_\text {eff}\nabla ^2w_0, \end{aligned}$$

hence the total fluorescence concentration recapitulates the behaviour of a pure diffusion system and the fluorescence recovery function is

84 $$\begin{aligned} F(t) = \mathscr {F}_S \left( \frac{r_n^2}{2 D_\text {eff} t} \right) . \end{aligned}$$

### Fisher information matrix

Here we derive the Fisher information Matrix from the $\eta \gg 1$ approximation (68). The eigenvalues of the matrix derived here are plotted in Fig. 2 for different values of $r_n$ and $\Delta t$. It is necessary to determine the partial derivatives with respect to each of the four model parameters, $D_u, D_v, k_\mathrm {on}, k_\mathrm {off}$. We begin by differentiating the function

85 $$\begin{aligned} \mathscr {F}_S(z) = e^{-z}(I_0(z) + I_1(z)). \end{aligned}$$

We will make use of identities,

86 $$\begin{aligned} \frac{\text {d}}{\text {d}z}I_0 (z) = I_1(z), \end{aligned}$$

and

87 $$\begin{aligned} \frac{\text {d}}{\text {d}z}I_\alpha (z) = I_{\alpha -1 } (z)+ \frac{\alpha }{z}I_{\alpha }(z) \therefore \frac{\text {d}}{\text {d}z}I_1 (z) = I_0 + \frac{1}{z}I_1(z). \end{aligned}$$

From (86) and (87) it follows that

88 $$\begin{aligned} \frac{\text {d}\mathscr {F}_S}{\text {d}z} = -\frac{e^{-z}I_1(z)}{z}. \end{aligned}$$

Then, taking $z=(r_n^2)(2Dt)^{-1}$ it takes a simple application of the chain rule to conclude that

89 $$\begin{aligned} \frac{\partial }{\partial D}\mathscr {F}_S\left( \frac{r_n^2}{2Dt}\right) = \frac{e^{-\frac{r_n^2}{2D t}} I_1\left( \frac{r_n^2}{2D t}\right) }{D}. \end{aligned}$$

It follows that

90 $$\begin{aligned} \frac{\partial F}{\partial D_u}= \frac{k_\mathrm {off}}{k_\mathrm {on}+k_\mathrm {off}} \frac{e^{-\frac{r_n^2}{2D_u t}} I_1\left( \frac{r_n^2}{2D_u t}\right) }{D_u}, \end{aligned}$$

and

91 $$\begin{aligned} \frac{\partial F}{\partial D_v}= \frac{k_\mathrm {on}}{k_\mathrm {on}+k_\mathrm {off}}e^{-k_\mathrm {off}t} \frac{e^{-\frac{r_n^2}{2D_v t}} I_1\left( \frac{r_n^2}{2D_v t}\right) }{D_v}. \end{aligned}$$

It follows from the quotient rule that

92 $$\begin{aligned} \frac{\partial F}{\partial k_\mathrm {on}}= \frac{k_\mathrm {off}e^{-k_\mathrm {off}t} \left( \mathscr {F}_S\left( \frac{r_n^2}{2D_v t} \right) +e^{k_\mathrm {off}t} - \mathscr {F}_S\left( \frac{r_n^2}{2D_u t}\right) e^{k_\mathrm {off}t} - 1 \right) }{(k_\mathrm {on}+k_\mathrm {off})^2}, \end{aligned}$$

and

93 $$\begin{aligned} \frac{\partial F}{\partial k_\mathrm {off}} = \frac{k_\mathrm {on}e^{-k_\mathrm {off}t} \left( \mathscr {F}_S\left( \frac{r_n^2}{2D_v t} \right) (1+k_\mathrm {on}t) +e^{k_\mathrm {off}t} -(k_\mathrm {on}+k_\mathrm {off})t - \mathscr {F}_S\left( \frac{r_n^2}{2D_u t}\right) e^{k_\mathrm {off}t} -1 \right) }{(k_\mathrm {on}+k_\mathrm {off})^2}. \end{aligned}$$

Now that we have explicitly derived the partial derivatives of *F* with respect to each of the four model parameters, from formula (14) in the main text it follows that

94 $$\begin{aligned} I_{i} = \begin{pmatrix} \left( \frac{\partial F}{\partial D_u}\right) ^2 &{} \frac{\partial F}{\partial D_u}\frac{\partial F}{\partial D_v} &{} \frac{\partial F}{\partial D_u}\frac{\partial F}{\partial k_\mathrm {on}} &{} \frac{\partial F}{\partial D_u}\frac{\partial F}{\partial k_\mathrm {off}} \\ \frac{\partial F}{\partial D_u}\frac{\partial F}{\partial D_v} &{} \left( \frac{\partial F}{\partial D_v}\right) ^2 &{} \frac{\partial F}{\partial D_v}\frac{\partial F}{\partial k_\mathrm {on}} &{} \frac{\partial F}{\partial D_v}\frac{\partial F}{\partial k_\mathrm {off}}\\ \frac{\partial F}{\partial D_u}\frac{\partial F}{\partial k_\mathrm {on}} &{} \frac{\partial F}{\partial D_v}\frac{\partial F}{\partial k_\mathrm {on}} &{} \left( \frac{\partial F}{\partial k_\mathrm {on}}\right) ^2 &{} \frac{\partial F}{\partial k_\mathrm {on}}\frac{\partial F}{\partial k_\mathrm {off}}\\ \frac{\partial F}{\partial D_u}\frac{\partial F}{\partial k_\mathrm {off}} &{} \frac{\partial F}{\partial D_v}\frac{\partial F}{\partial k_\mathrm {off}} &{} \frac{\partial F}{\partial k_\mathrm {on}}\frac{\partial F}{\partial k_\mathrm {off}} &{}\left( \frac{\partial F}{\partial k_\mathrm {off}}\right) ^2 \\ \end{pmatrix}(t_i). \end{aligned}$$

Then the Fisher information matrix is simply

95 $$\begin{aligned} I=\sum _i \frac{1}{\sigma _i^2}I_i. \end{aligned}$$
